# Design, synthesis, and pharmacological evaluation of 2-amino-5-nitrothiazole derived semicarbazones as dual inhibitors of monoamine oxidase and cholinesterase: effect of the size of aryl binding site

**DOI:** 10.1080/14756366.2017.1389920

**Published:** 2017-11-03

**Authors:** Rati K. P. Tripathi, Vishnu M. Sasi, Sukesh K. Gupta, Sairam Krishnamurthy, Senthil R. Ayyannan

**Affiliations:** a Pharmaceutical Chemistry Research Laboratory, Department of Pharmaceutical Engineering & Technology, Indian Institute of Technology (Banaras Hindu University), Varanasi, India;; b Neurotherapeutics Research Laboratory, Department of Pharmaceutical Engineering & Technology, Indian Institute of Technology (Banaras Hindu University), Varanasi, India

**Keywords:** 2-Amino-5-nitrothiazole, monoamine oxidase, Cholinesterase, dual inhibitors, molecular docking

## Abstract

A series of 2-amino-5-nitrothiazole derived semicarbazones were designed, synthesised and investigated for MAO and ChE inhibition properties. Most of the compounds showed preferential inhibition towards MAO-B. Compound **4**, (1-(1-(4-Bromophenyl)ethylidene)-4-(5-nitrothiazol-2-yl)semicarbazide) emerged as lead candidate (IC_50_ = 0.212 µM, SI = 331.04) against MAO-B; whereas compounds **21** 1-(5-Bromo-2-oxoindolin-3-ylidene)-4-(5-nitrothiazol-2-yl)semicarbazide (IC_50_ = 0.264 µM) and **17** 1-((4-Chlorophenyl) (phenyl)methylene)-4-(5-nitrothiazol-2-yl)semicarbazide (IC_50_ = 0.024 µM) emerged as lead AChE and BuChE inhibitors respectively; with activity of compound **21** almost equivalent to tacrine. Kinetic studies indicated that compound **4** exhibited competitive and reversible MAO-B inhibition while compounds **21** and **17** showed mixed-type of AChE and BuChE inhibition respectively. Docking studies revealed that these compounds were well-accommodated within MAO-B and ChE active sites through stable hydrogen bonding and/or hydrophobic interactions. This study revealed the requirement of small heteroaryl ring at amino terminal of semicarbazone template for preferential inhibition and selectivity towards MAO-B. Our results suggest that 5-nitrothiazole derived semicarbazones could be further exploited for its multi-targeted role in development of anti-neurodegenerative agents.

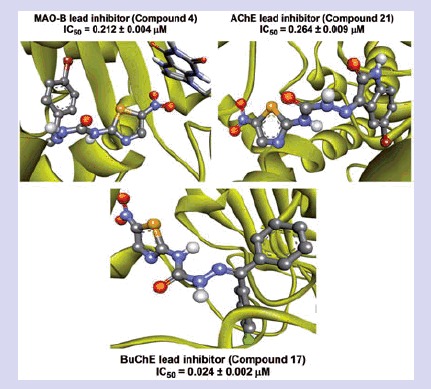

A library of 2-amino-5-nitrothiazole derived semicarbazones (**4**–**21**) was designed, synthesised and evaluated for *in vitro* MAO and ChE inhibitory activity. Compounds **4**, **21** and **17** (shown) have emerged as lead MAO-B (IC_50_:0.212 µM, competitive and reversible), AChE (IC_50_:0.264 µM, mixed and reversible) and BuChE (IC_50_:0.024 µM, mixed and reversible) inhibitor respectively. SAR studies disclosed several structural aspects significant for potency and selectivity and indicated the role of size of aryl binding site in potency and selectivity towards MAO-B. Antioxidant activity and neurotoxicity screening results further suggested their multifunctional potential for the therapy of neurodegenerative diseases.

## Introduction

The present growth in the state-of-art medical technologies has resulted in the augmentation in the average life expectancy, particularly among the elderly population, the consequences of which include social and complex issues like neurodegenerative diseases (NDDs). NDDs, the umbrella term used for a wide range of progressive neurological disorders, is highly linked to injuries caused in the brain neurons from which the recovery is very difficult or negligible. Selective neuronal loss in particular brain regions triggers diverse categories of NDDs such as Alzheimer’s disease (AD), Parkinson’s disease (PD), depressive illness, stroke and many others. Among these, the two most common types are AD and PD, and fundamentally, till date, no permanent clinical cure is available for these diseases^1^. And for this reason, modification of the disease path by means of neuroprotective therapy is an essential unmet clinical necessity.

Although the aetiology of NDDs is not completely known, oxidative stress and low levels of acetylcholine (ACh) seem to play significant roles^2^. Monoamine oxidase B (MAO-B) activity is also reported to be increased in association with gliosis, which can result in higher levels of hydrogen peroxide and oxidative stress for vulnerable neurons affected by NDDs^3^. Anti-acetylcholinesterase (anti-AChE) agents such as tacrine and donepezil have shown a modest improvement in memory and cognitive function but do not appear to prevent or slow the progressive neurodegeneration^4^. On the other hand, rasagiline, a selective MAO-B inhibitor, has been reported to retard the further deterioration of cognitive functions, displaying neuroprotective activity^5^. Because of the complex and multifactorial nature of NDDs and diverse cerebral mechanisms implicated in their treatment, it is unlikely that a monotherapy will provide a comprehensive and satisfactory therapeutic solution. Such therapy is more likely to be achieved by the use of multitarget-directed ligands (MTDLs) that incorporate several pharmacological traits into a single molecular entity and work in a synergistic manner. During the last decade, the emerging MTDL strategy yielded several promising multifunctional drug candidates for the treatment of NDDs^6–8^. Particularly, the MTDL strategy targeting monoamine oxidases (MAO-A and MAO-B) and cholinesterases (ChE) namely acetylcholinesterase (AChE) and butyrylcholinesterase (BuChE)) represents one of the promising approaches due to their validated neuroprotective, neurorestorative and cognition enhancing capability in addition to their effect on monoaminergic neurotransmission^6^^,^^8^. Attempts to combine anti-MAO and anti-ChE activities in one molecular entity have previously been reported^9–13^.

Monoamine oxidases (MAOs) are a family of flavin adenine dinucleotide (FAD) containing enzymes present in the outer membrane of mitochondria in various cells found in nerve terminals, liver, gut mucosa and other tissues^14^ and are responsible for catalysing the oxidative deamination of biogenic monoamines and neurotransmitters^15^^,^^16^. Based on their substrate and inhibitor specificities^17–19^, MAOs exist in two enzymatic isoforms: MAO-A and MAO-B and contributes to about 70% amino acid homologous identity They are encoded by different genes located on the X chromosome^20–22^. MAO-A mostly metabolises serotonin (5-HT), noradrenaline and adrenaline and are selectively inhibited by clorgyline, while MAO-B generally oxidises benzylamine and β-phenylethylamine (PEA) and is inhibited selectively by selegiline. Selective inhibitors of MAO-A have been shown to be effective against depression and anxiety^23^^,^^24^, whereas selective MAO-B inhibitors are useful in the treatment of several NDDs such as AD^25^^,^^26^ and PD^27–29^. Furthermore, NDDs are commonly associated with depressive symptoms^30^. In addition, elevated levels of monoamine oxidases in the neuronal tissue results in the production of by-product, hydrogen peroxide, which further causes increase in the level of reactive oxygen species (ROS) playing a major role in the pathogenesis of NDDs. Consequently, MAO inhibitors are regarded as potential drug candidates for NDDs due to their ability to inhibit oxidative damage^31^.

Acetylcholinesterase (AChE) belongs to serine hydrolase family of enzymes catalysing the hydrolysis of neurotransmitter acetylcholine (ACh) into choline and acetic acid, consequently causing cessation of cholinergic neurotransmission^32^. AChE is widely distributed in conducting tissues like nerves and muscles, central and peripheral tissues, motor and sensory fibres in addition to cholinergic and non-cholinergic fibres^33^. It is also present in plasma and blood cells^34^^,^^35^. Inhibition of the cleavage of acetylcholine by anti-acetylcholinesterase agents elevates the level of acetylcholine (ACh) in cholinergic synapses and can consequently influence various pathogenic processes and in that way affords a potential therapy for AD^36^. Besides its role in cholinergic neurotransmission, it is also implicated in non-cholinergic responses like cell differentiation and proliferation, stress and amyloid formation^37^. In addition to AChE, the enzyme butyrylcholinesterase (BuChE) is important in the regulation of cholinergic system and it is reported to efficiently catalyse the hydrolysis of acetylcholine^38^. BuChE is expressed in distinct population of neurons, some of which contain AChE. The importance of BuChE is further supported by the observation that AChE-knockout mice survive to adulthood indicating that BuChE is able to compensate for the lack of AChE, allowing the continued regulation of cholinergic neurotransmission^39^. At present, only five anti-ChE agents exist which includes donepezil, tacrine, galantamine, rivastigmine, and ethopropazine. Because of the limited number of clinically existing ChE inhibitors, exploration for novel ChE inhibitors is of great interest worldwide.

The discovery of crystal structures of both the human MAO isozymes (hMAO-A and hMAO-B) and human recombinant AChE (rhAChE) and human butyrylcholinesterase (BuChE) enzyme, alone and in complex with various inhibitors has brought an important breakthrough in the development of small molecule inhibitors against these enzymes^40–44^. Also this structure-guided approach has imparted a better understanding of the pharmacophoric elements for the rational design of dual inhibitors of MAO and ChE^45^^,^^46^.

**Figure 1.**
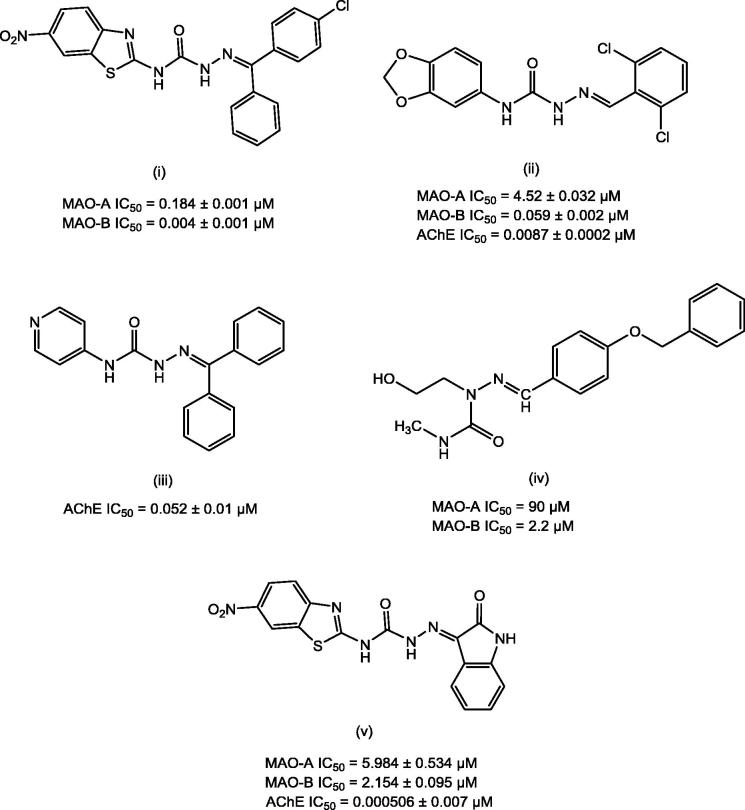
Lead MAO/AChE inhibitors possessing semicarbazone scaffold.

Semicarbazones (SCZs) have been documented as promising scaffolds with multitude of pharmacological actions including MAO and AChE inhibitory properties^47–50^. Figure 1 illustrates some of the lead MAO and AChE inhibitors bearing semicarbazone scaffold.

**Figure 2.**
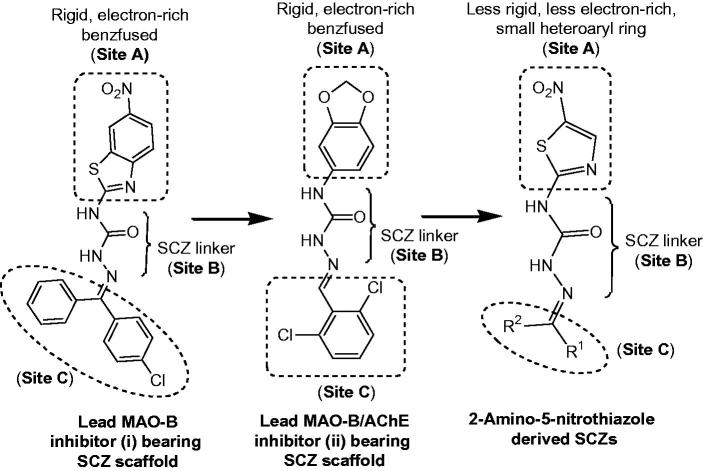
Design of semicarbazones possessing 5-nitrothiazole moiety and other essential pharmacophoric elements.

With a view to recognise novel heterocyclic scaffold and in continuation to our work on SCZs, we designed a series of **18** semicarbazones (Figure 2) with the objective of investigating the effect of size of aryl ring at site A (Figure 2) on the MAO/ChE inhibitory activity. Thus, the large hydrophobic heteroaryl moiety 6-nitrobenzothiazole (i)^49^ or 3,4-(methylenedioxy)phenyl (ii)^50^ at site A was replaced with a relatively less rigid, less electron-rich, small heteroaryl ring, 5-nitrothiazole (Figure 2). While at site C, the imino terminal of semicarbazone template, (un)substituted aryl or herteroaryl moieties with varying degree of hydrophobicity were incorporated for deriving the structure–activity relationships.

Herein we report the investigation of 5-nitrothiazole derived SCZs as new multi-target-directed ligands (MTDLs) aimed to be effective against NDDs. The pharmacological evaluations of the synthesised 5-nitrothiazole derived semicarbazones included the *in vitro* MAO-A/MAO-B inhibition and *in vitro* AChE/BuChE inhibition assays. Kinetic and reversibility studies were performed to explore the mode of inhibition of the most active inhibitors against MAO-A, MAO-B, AChE and BuChE. Further, molecular docking simulations were accomplished to identify the binding site, orientation and interactions of MAO/ChE inhibitors within their respective enzyme active sites using AutoDock 4.2 in addition to the determination of the free energies of binding (*Δ*G) and inhibition constants (*K*_i_) of the experimentally tested compounds. Additionally, the synthesised compounds were tested for their antioxidant potential by *in vitro* DPPH radical scavenging assay. Neurotoxicity screening was performed for the selected compounds using rotarod apparatus.

## Materials and methods

### Chemistry

Starting materials and reagents were procured from commercial suppliers Sigma-Aldrich and Merck and were used without further purification. The progress of the reactions was monitored using thin-layer chromatography. Melting points were determined by one end open capillary tubes on a Sonar melting point apparatus and are uncorrected. IR spectra of intermediates and final compounds were recorded as potassium bromide pellets on Shimadzu FT-IR 8400S infrared spectrophotometer. Dry solvents were used throughout. ^1^H and ^13^C NMR spectra were recorded on a Jeol AL300 FT-NMR spectrometer at the operating frequency of 300 and 75 MHz, respectively. All the NMR measurements were conducted in (D_6_)DMSO and tetramethylsilane (TMS) was used as an internal reference. Chemical shifts (*δ*) were reported in parts per million (ppm) using the residual solvent line as the internal standard. Coupling constants *J* were expressed in hertz (Hz). The exchangeable protons were confirmed by the addition of D_2_O. The mass spectra were measured on a Thermo LCQ Advantage Max Ion Trap Mass spectrometer. Elemental analyses (C, H, N) were undertaken with Exeter Analytical Inc. Model CE-440 CHN analyser.

### Synthesis of intermediates

*Procedure for the preparation of 1-(5-nitrothiazol-2-yl)urea****2***: 2-Amino-5-nitrothiazole **1** (0.030 mol) was dissolved in glacial acetic acid (30 ml) with continuous stirring on a magnetic stirrer. To this, a warm solution of sodium cyanate (0.031 mol, 2.0 equiv) in H_2_O (60 ml) was added with vigorous stirring. The mixture was stirred for 5 h before being left to stand overnight. The resultant solid was collected by filtration, washed with ice cold water to remove excess glacial acetic acid and dried. The reddish-brown coloured crude product 1-(5-nitrothiazol-2-yl)urea **2** obtained was then recrystallised from 95% ethanol.

*1-(5-nitrothiazol-2-yl)urea****2****:* IR (KBr): ʋ = 3402.54, 3254.02 (N–H str), 1705.13 (C=O str), 1622.19 (C=N str), 1489.10, 1371.3 (NO_2_ str), 1201.69 (C–N str); ^1^H NMR ((D_6_)DMSO, D_2_O exchange, 300 MHz): *δ* = 8.42 (s, 1H, thiazole C–H), 8.59 (s, 1H, NH), 9.02 ppm (s, 2H, NH_2_); ^13^C NMR ((D_6_)DMSO, 75 MHz): *δ* = 107.70 (thiazole C-5), 143.92 (thiazole C-4), 158.93 (C=O), 160.51 ppm (thiazole C-2).

*Procedure for the preparation of 4-(5-nitrothiazol-2-yl)semicarbazide****3****:* In the RB flask containing compound **2** (0.03 mol) dissolved in ethanol (30 ml), hydrazine hydrate (0.03 mol, 2.0 equiv) was added and the reaction mixture was refluxed for about 18 h. Solvent was evaporated, and the resultant residue obtained was recrystallised from 95% ethanol.

*4-(5-nitrothiazol-2-yl)semicarbazide****3****:* IR (KBr): ʋ = 3313.82, 3178.79 (N–H str), 1662.69 (C=O str), 1541.18 (C=N str), 1508.38, 1357.93 (NO_2_ str), 1276.92 (C–N str); ^1^H NMR ((D_6_)DMSO, D_2_O exchange, 300 MHz): *δ* = 1.95 (s, 2H, NH_2_), 8.49 (s, 1H, thiazole C–H), 9.132 (s, 1H, NH), 10.63 ppm (s, 1H, CONH); ^13^C NMR ((D_6_)DMSO, 75 MHz): *δ* = 107.95 (thiazole C-5), 142.06 (thiazole C-4), 158.91 (C=O), 162.84 ppm (thiazole C-2).

### Synthesis of final compounds

*General procedure for the preparation of compounds****4****–****21****:* The final compounds **4**–**21** (substituted semicarbazones) were synthesised by the reaction of compound **3** (0.003 mol) with appropriate aldehydes, ketones or 5-(un)substituted isatin (0.003 mol). The reaction mixture was adjusted to pH 5–6 by adding few drops of glacial acetic acid and refluxed for 29–80 h. The solvent was either evaporated or the contents of the flask was quenched in ice cold water and the crude product obtained was filtered, dried and recrystallised from 95% ethanol to produce final compounds **4**–**21**.

*1-(1-(4-Bromophenyl)ethylidene)-4-(5-nitrothiazol-2-yl)semicarbazide****4****:* IR (KBr): ʋ = 3410.26, 3156 (N–H str), 3082.35 (aromatic C–H str), 1683.93 (C=O str), 1624.42 (C=N str), 1586.25, 1381.08 (NO_2_ str), 1300.07 (C–N str), 713.89 (C–Br str); ^1^H NMR ((D_6_)DMSO, D_2_O exchange, 300 MHz): *δ* = 1.14 (s, 3H, CH_3_), 7.50 (d, *J* = 6.3 Hz, 2H, Ar C-3, Ar C-5), 7.78 (d, *J* = 5.7 Hz, 2H, Ar C-2, Ar C-6), 8.81 (s, 1H, thiazole C–H), 9.17 (s, 1H, NH), 10.48 ppm (s, 1H, CONH); ^13^C NMR ((D_6_)DMSO, 75 MHz): *δ* = 19.14 (CH_3_), 107.85 (thiazole C-5), 128.05 (Ar C-4), 129.58 (Ar C-2, Ar C-6), 130.90 (Ar C-3, Ar C-5), 133.33 (Ar C-1), 140.88 (thiazole C-4), 156.11 (C=O), 164.25 (thiazole C-2), 168.95 ppm (C=N); Anal. for C_12_H_10_BrN_5_O_3_S: calcd: C 37.51, H 2.62, N 18.23, found: C 37.49, H 2.65, N 18.30.

*1-(1-(4-Chlorophenyl)ethylidene)-4-(5-nitrothiazol-2-yl)semicarbazide****5****:* IR (KBr): ʋ = 3431.48, 3313.82 (N–H str), 3090.07 (aromatic C–H str), 1670.41 (C=O str), 1606.76 (C=N str), 1531.30, 1400.27 (NO_2_ str), 1161.39 (C–N str), 831.35 (C–Cl str); ^1^H NMR ((D_6_)DMSO, D_2_O exchange, 300 MHz): *δ* = 1.13 (s, 3H, CH_3_), 7.54 (d, *J* = 6.6 Hz, 2H, Ar C-3, Ar C-5), 7.85 (d, *J* = 6.6 Hz, 2H, Ar C-2, Ar C-6), 8.29 (s, 1H, thiazole C–H), 9.08 (s, 1H, NH), 10.33 ppm (s, 1H, CONH); ^13^C NMR ((D_6_)DMSO, 75 MHz): *δ* = 19.12 (CH_3_), 109.17 (thiazole C-5), 129.92 (Ar C-3, Ar C-5), 131.54 (Ar C-2, Ar C-6), 132.15 (Ar C-1), 134.13 (Ar C-4), 140.65 (thiazole C-4), 158.54 (C=O), 163.93 (thiazole C-2), 171.72 ppm (C=N); Anal. for C_12_H_10_ClN_5_O_3_S: calcd: C 42.42, H 2.97, N 20.61, found: C 42.36, H 3.01, N 20.56.

*1-(1-(4-Fluorophenyl)ethylidene)-4-(5-nitrothiazol-2-yl)semicarbazide****6****:* IR (KBr): ʋ = 3419.90, 3306.10 (N–H str), 3064.99 (aromatic C–H str), 1699.77 (C=O str), 1581.68 (C=N str), 1558.54, 1410.01 (NO_2_ str), 1232.55 (C–N str), 1012.66 (C–F str); ^1^H NMR ((D_6_)DMSO, 300 MHz): *δ* = 1.12 (s, 3H, CH_3_), 7.28 (d, *J* = 6.6 Hz, 2H, Ar C-3, Ar C-5), 7.92 (d, *J* = 5.7 Hz, 2H, Ar C-2, Ar C-6), 8.39 (s, 1H, thiazole C–H), 8.98 (s, 1H, NH), 10.14 ppm (s, 1H, CONH); ^13^C NMR ((D_6_)DMSO, 75 MHz): *δ* = 19.16 (CH_3_), 109.85 (thiazole C-5), 116.51 (Ar C-3, Ar C-5), 127.62 (Ar C-1), 133.03 (Ar C-2, Ar C-6), 140.63 (thiazole C-4), 156.94 (C=O), 164.75 (thiazole C-2), 166.25 (Ar C-4), 168.78 ppm (C=N); Anal. for C_12_H_10_FN_5_O_3_S: calcd: C 44.58, H 3.12, N 21.66, found: C 44.53, H 3.15, N 21.61.

*1-(1-(4-Hydroxyphenyl)ethylidene)-4-(5-nitrothiazol-2-yl)semicarbazide****7****:* IR (KBr): ʋ = 3520.21 (O–H str), 3356.25, 3159.51 (N–H str), 2997.48 (aromatic C–H str), 1670.41 (C=O str), 1606.76 (C=N str), 1550.82, 1442.80 (NO_2_ str), 1224.84 (C–N str); ^1^H NMR ((D_6_)DMSO, 300 MHz): *δ* = 1.03 (s, 3H, CH_3_), 4.99 (s, 1H, OH), 7.04 (d, *J* = 5.1 Hz, 2H, Ar C-3, Ar C-5), 7.68 (d, *J* = 5.7 Hz, 2H, Ar C-2, Ar C-6), 8.43 (s, 1H, thiazole C–H), 9.09 (s, 1H, NH), 10.25 ppm (s, 1H, CONH); ^13^C NMR ((D_6_)DMSO, 75 MHz): *δ* = 19.31 (CH_3_), 109.77 (thiazole C-5), 117.63 (Ar C-3, Ar C-5), 128.92 (Ar C-1), 132.30 (Ar C-2, Ar C-6), 142.39 (thiazole C-4), 156.75 (C=O), 161.86 (Ar C-4), 164.36 (thiazole C-2), 168.71 ppm (C=N); Anal. for C_12_H_11_N_5_O_4_S: calcd: C 44.86, H 3.45, N 21.80, found: C 44.82, H 3.48, N 21.78.

*1-(1-(4-Nitrophenyl)ethylidene)-4-(5-nitrothiazol-2-yl)semicarbazide****8****:* IR (KBr): ʋ = 3487.42, 3331.18 (N–H str), 3111.28 (aromatic C–H str), 1695.49 (C=O str), 1585.54 (C=N str), 1508.38, 1338.64 (NO_2_ str), 1107.18 (C–N str); ^1^H NMR ((D_6_)DMSO, 300 MHz): *δ* = 2.22 (s, 3H, CH_3_), 8.28 (d, *J* = 8.1 Hz, 2H, Ar C-3, Ar C-5), 8.14 (d, *J* = 8.2 Hz, 2H, Ar C-2, Ar C-6), 8.85 (s, 1H, thiazole C–H), 9.34 (s, 1H, NH), 9.75 ppm (s, 1H, CONH); ^13^C NMR ((D_6_)DMSO, 75 MHz): *δ* = 19.03 (CH_3_), 107.94 (thiazole C-5), 127.85 (Ar C-3, Ar C-5), 129.52 (Ar C-2, Ar C-6), 140.76 (thiazole C-4), 143.30 (Ar C-1), 148.08 (Ar C-4), 156.04 (C=O), 164.24 (thiazole C-2), 169.60 ppm (C=N); MS: *m/z* = 351.39 (M + 1)^+^; Anal. for C_12_H_10_N_6_O_5_S: calcd: C 41.14, H 2.88, N 23.99, found: C 41.17, H 2.86, N 24.05.

*1-(2-Bromo-1-(4-bromophenyl)ethylidene)-4-(5-nitrothiazol-2-yl)semicarbazide****9****:* IR (KBr): ʋ = 3410.26, 3240.52 (N–H str), 3005.20 (aromatic C–H str), 2895.20 (CH_2_ str), 1697.41 (C=O str), 1587.47 (C=N str), 1508.38, 1458.23 (NO_2_ str), 1232.55 (C–N str), 831.35 (C–Br str); ^1^H NMR ((D_6_)DMSO, 300 MHz): *δ* = 3.56 (s, 2H, CH_2_), 8.14 (d, *J* = 7.2 Hz, 2H, Ar C-2, Ar C-6), 8.19 (s, 1H, thiazole C–H), 8.28 (d, *J* = 8.1 Hz, 2H, Ar C-3, Ar C-5), 9.27 (s, 1H, NH), 10.08 ppm (s, 1H, CONH); ^13^C NMR ((D_6_)DMSO, 75 MHz): *δ* = 74.77 (CH_2_), 109.85 (thiazole C-5), 116.51 (Ar C-3, Ar C-5), 127.62 (Ar C-1), 133.03 (Ar C-2, Ar C-6), 140.63 (thiazole C-4), 156.94 (C=O), 166.25 (Ar C-4), 164.75 (thiazole C-2), 168.78 ppm (C=N); Anal. for C_12_H_9_Br_2_N_5_O_3_S: calcd: C 31.12, H 1.96, N 15.12, found: C 31.09, H 1.93, N 15.20.

*1-(4-Bromobenzylidene)-4-(5-nitrothiazol-2-yl)semicarbazide****10****:* IR (KBr): ʋ = 3443.05, 3281.02 (N–H str), 3086.25 (aromatic C–H str), 1662.69 (C=O str), 1591.33 (C=N str), 1556.61, 1410.01 (NO_2_ str), 1273.06 (C–N str), 798.56 (C–Br str); ^1^H NMR ((D_6_)DMSO, D_2_O exchange, 300 MHz): *δ* = 7.67 (d, *J* = 7.2 Hz, 2H, Ar C-2, Ar C-6), 7.85 (d, *J* = 7.5 Hz, 2H, Ar C-3, Ar C-5), 8.19 (s, 1H, CH), 8.26 (s, 1H, thiazole C–H), 9.68 (s, 1H, NH), 10.05 ppm (s, 1H, CONH); ^13^C NMR ((D_6_)DMSO, 75 MHz): *δ* = 109.50 (thiazole C-5), 124.55 (Ar C-4), 128.77 (Ar C-2, Ar C-6), 129.81 (Ar C-3, Ar C-5), 132.63 (Ar C-1), 141.02 (thiazole C-4), 156.71 (C=N), 158.77 (C=O), 164.22 ppm (thiazole C-2); Anal. for C_11_H_8_BrN_5_O_3_S: calcd: C 35.69, H 2.18, N 18.92, found: C 35.62, H 2.23, N 18.99.

*1-(4-Hydroxybenzylidene)-4-(5-nitrothiazol-2-yl)semicarbazide****11****:* IR (KBr): ʋ = 3419.66 (OH str), 3325.39, 3192.30 (N–H str), 2931.90 (aromatic C–H str), 1670.41 (C=O str), 1599.04 (C=N str), 1458.23, 1388.79 (NO_2_ str), 1161.19 (C–N str); ^1^H NMR ((D_6_)DMSO, 300 MHz): *δ* = 4.96 (s, 1H, OH), 6.85 (d, *J* = 8.4 Hz, 2H, Ar C-3, Ar C-5), 7.42 (d, *J* = 7.5 Hz, 2H, Ar C-2, Ar C-6), 8.09 (s, 1H, CH), 8.58 (s, 1H, thiazole C–H), 9.68 (s, 1H, NH), 10.08 ppm (s, 1H, CONH); ^13^C NMR ((D_6_)DMSO, 75 MHz): *δ* = 109.52 (thiazole C-5), 115.75 (Ar C-3, Ar C-5), 121.12 (Ar C-1), 122.54 (Ar C-2, Ar C-6), 141.07 (thiazole C-4), 144.66 (C=N), 158.33 (C=O), 161.09 (Ar C-4), 164.26 ppm (thiazole C-2); Anal. for C_11_H_9_N_5_O_4_S: calcd: C 43.00, H 2.95, N 22.79, found: C 43.09, H 2.99, N 22.73.

*1-(2,3-Dichlorobenzylidene)-4-(5-nitrothiazol-2-yl)semicarbazide****12****:* IR (KBr): ʋ = 3448.84, 3252.09 (N–H str), 2995.55 (aromatic C–H str), 1654.98 (C=O str), 1560.46 (C=N str), 1469.66, 1383.64 (NO_2_ str), 1101.39 (C–N str), 742.62 (C–Cl str); ^1^H NMR ((D_6_)DMSO, D_2_O exchange, 300 MHz): *δ* = 7.49 (dd, *J* = 7.2, 6.5 Hz, 1H, Ar C-5), 7.61 (d, *J* = 6.6 Hz, 1H, Ar C-4), 7.86 (d, *J* = 6.9 Hz, 1H, Ar C-6), 8.30 (s, 1H, CH), 8.85 (s, 1H, thiazole C–H), 9.21 (s, 1H, NH), 10.25 ppm (s, 1H, CONH); ^13^C NMR ((D_6_)DMSO, 75 MHz): *δ* = 107.89 (thiazole C-5), 111.73 (Ar C-5), 128.70 (Ar C-6), 128.83 (Ar C-4), 133.79 (Ar C-3), 134.80 (Ar C-2), 136.87 (Ar C-1), 142.59 (thiazole C-4), 146.90 (C=N), 158.80 (C=O), 167.88 ppm (thiazole C-2); Anal. for C_11_H_7_Cl_2_N_5_O_3_S: calcd: C 36.68, H 1.96, N 19.44, found: C 36.62, H 1.99, N 19.49.

*1-(2,4-Dichlorobenzylidene)-4-(5-nitrothiazol-2-yl)semicarbazide****13****:* IR (KBr): ʋ = 3400.09, 3346.61 (N–H str), 3088.14 (aromatic C–H str), 1680.05 (C=O str), 1585.54 (C=N str), 1467.88, 1381.08 (NO_2_ str), 1199.76 (C–N str), 823.63 (C–Cl str); ^1^H NMR ((D_6_)DMSO, 300 MHz): *δ* = 6.92 (d, *J* = 7.5 Hz, 1H, Ar C-5), 7.02 (s, 1H, Ar C-3), 7.41 (d, *J* = 6.0 Hz, 1H, Ar C-6), 8.11 (s, 1H, CH), 8.68 (s, 1H, thiazole C–H), 9.23 (s, 1H, NH), 10.99 ppm (s, 1H, CONH); ^13^C NMR ((D_6_)DMSO, 75 MHz): *δ* = 109.16 (thiazole C-5), 129.92 (Ar C-5), 130.23 (Ar C-3), 130.94 (Ar C-1), 131.08 (Ar C-6), 137.32 (Ar C-2), 139.15 (Ar C-4), 139.71 (thiazole C-4), 148.51 (C=N), 158.65 (C=O), 162.98 ppm (thiazole C-2); **MS:***m/z* = 361.14 (M + 1)^+^; Anal. for C_11_H_7_Cl_2_N_5_O_3_S: calcd: C 36.68, H 1.96, N 19.44, found: C 36.60, H 1.98, N 19.47.

*1-(2,6-Dichlorobenzylidene)-4-(5-nitrothiazol-2-yl)semicarbazide****14****:* IR (KBr): ʋ = 3446.91, 3369.75 (N–H str), 3064.99 (aromatic C–H str), 1658.84 (C=O str), 1624.12 (C=N str), 1556.61, 1427.37 (NO_2_ str), 1190.12 (C–N str), 777.34 (C–Cl str); ^1^H NMR ((D_6_)DMSO, D_2_O exchange, 300 MHz): *δ* = 7.47 (d, *J* = 6.6 Hz, 3H, Ar C-3, Ar C-4, Ar C-5), 8.22 (s, 1H, CH), 8.58 (s, 1H, thiazole C–H), 9.17 (s, 1H, NH), 10.05 ppm (s, 1H, CONH); ^13^C NMR ((D_6_)DMSO, 75 MHz): *δ* = 109.25 (thiazole C-5), 129.11 (Ar C-3, Ar C-5), 130.71 (Ar C-1), 135.70 (Ar C-4), 138.48 (Ar C-2, Ar C-6), 141.29 (thiazole C-4), 144.35 (C=N), 158.72 (C=O), 162.97 ppm (thiazole C-2); Anal. for C_11_H_7_Cl_2_N_5_O_3_S: calcd: C 36.68, H 1.96, N 19.44, found: C 36.74, H 2.03, N 19.39.

*1-(2,5-Dimethoxybenzylidene)-4-(5-nitrothiazol-2-yl)semicarbazide****15****:* IR (KBr): ʋ = 3524.06, 3342.75 (N–H str), 3091.99 (aromatic C–H str), 1660.77 (C=O str), 1564.32 (C=N str), 1413.87, 1373.36 (NO_2_ str), 1149.61 (C–N str); 1116.82 (C–O–C str); ^1^H NMR ((D_6_)DMSO, 300 MHz): *δ* = 3.36 (s, 6H, OCH_3_), 6.85 (d, *J* = 7.5 Hz, 2H, Ar C-3, Ar C-5), 7.26 (s, 1H, Ar C-6), 8.19 (s, 1H, CH), 8.36 (s, 1H, thiazole C–H), 9.90 (s, 1H, NH), 10.69 ppm (s, 1H, CONH); ^13^C NMR ((D_6_)DMSO, 75 MHz): *δ* = 59.93 (OCH_3_), 109.16 (thiazole C-5), 117.45 (Ar C-4), 118.26 (Ar C-6), 119.76 (Ar C-3), 121.54 (Ar C-1), 142.12 (thiazole C-4), 145.65 (C=N), 148.53 (Ar C-2), 149.81 (Ar C-5), 158.31 (C=O), 163.23 ppm (thiazole C-2); Anal. for C_13_H_13_N_5_O_5_S: calcd: C 44.44, H 3.73, N 19.93, found: C 44.49, H 3.64, N 19.98.

*1-(Diphenylmethylene)-4-(5-nitrothiazol-2-yl)semicarbazide****16****:* IR (KBr): ʋ = 3428.19, 3358.70 (N–H str), 3078.49 (aromatic C–H str), 1660.81 (C=O str), 1589.47 (C=N str), 1556.52, 1431.89 (NO_2_ str); ^1^H NMR ((D_6_)DMSO, D_2_O exchange, 300 MHz): *δ* = 6.85 (dd, *J* = 7.2, 6.7 Hz, 3H, Ar C-3, Ar C-4, Ar C-5), 7.02 (dd, *J* = 6.9, 5.6 Hz, 3H, Ar’ C-3, Ar’ C-4, Ar’ C-5), 7.24 (d, *J* = 7.2 Hz, 2H, Ar’ C-2, Ar’ C-6), 7.94 (d, *J* = 6.9 Hz, 2H, Ar C-2, Ar C-6), 8.39 (s, 1H, thiazole C–H), 9.88 (s, 1H, NH), 10.19 ppm (s, 1H, CONH); ^13^C NMR ((D_6_)DMSO, 75 MHz): *δ* = 110.24 (thiazole C-5), 126.31 (Ar C-3, Ar C-5),130.29 (Ar C-2, Ar C-6), 132.63 (Ar C-1), 133.11 (Ar C-4), 140.11 (thiazole C-4), 156.45 (C=N), 158.43 (C=O), 163.33 ppm (thiazole C-2); Anal. for C_17_H_13_N_5_O_3_S: calcd: C 55.58, H 3.57, N 19.06, found: C 55.52, H 3.61, N 19.14.

*1-((4-Chlorophenyl)(phenyl)methylene)-4-(5-nitrothiazol-2-yl)semicarbazide****17****:* IR (KBr): ʋ = 3443.05, 3289.25 (N–H str), 3054.89 (aromatic C–H str), 1647.26 (C=O str), 1590.61 (C=N str), 1560.46, 1437.02 (NO_2_ str), 1195.30 (C–N str), 781.20 (C–Cl str); ^1^H NMR ((D_6_)DMSO, 300 MHz): *δ* = 7.67 (d, *J* = 6.9 Hz, 2H, Ar C-3, Ar C-5), 7.78 (d, *J* = 7.2 Hz, 2H, Ar’ C-2, Ar’ C-6), 7.94 (d, *J* = 7.5 Hz, 2H, Ar C-2, Ar C-6), 8.17 (dd, *J* = 7.2, 5.8 Hz, 3H, Ar’ C-3, Ar’ C-4, Ar’ C-5), 8.89 (s, 1H, thiazole C–H), 9.16 (s, 1H, NH), 10.28 ppm (s, 1H, CONH); ^13^C NMR ((D_6_)DMSO, 75 MHz): *δ* = 110.58 (thiazole C-5), 126.78 (Ar’ C-3, Ar’ C-5), 128.32 (Ar C-3, Ar C-5), 129.13 (Ar’ C-2, Ar’ C-6), 131.56 (Ar C-2, Ar C-6), 132.11 (Ar C-1), 133.53 (Ar’ C-4), 135.32 (Ar’ C-1), 137.13 (Ar C-4), 138.66 (thiazole C-4), 156.61 (C=N), 158.30 (C=O), 163.12 ppm (thiazole C-2); Anal. for C_17_H_12_ClN_5_O_3_S: calcd: C 50.81, H 3.01, N 17.43, found: C 50.88, H 3.05, N 17.40.

*1-((4-Hydroxyphenyl)(phenyl)methylene)-4-(5-nitrothiazol-2-yl)semicarbazide****18****:* IR (KBr): ʋ = 3427.62 (OH str), 3300.31, 3167.22 (N–H str), 2955.04 (aromatic C–H str), 1654.84 (C=O str), 1600.97 (C=N str), 1560.46, 1444.73 (NO_2_ str), 1288.49 (C–N str); ^1^H NMR ((D_6_)DMSO, 300 MHz): *δ* = 4.92 (s, 1H, OH), 7.59 (d, *J* = 6.6 Hz, 2H, Ar’ C-2, Ar’ C-6), 7.83 (d, *J* = 8.7 Hz, 2H, Ar C-2, Ar C-6), 7.97 (d, *J* = 7.5 Hz, 2H, Ar C-3, Ar C-5), 8.09 (dd, *J* = 8.4, 6.3 Hz, 3H, Ar’ C-3, Ar’ C-4, Ar’ C-5), 8.85 (s, 1H, thiazole C–H), 9.66 (s, 1H, NH), 10.31 ppm (s, 1H, CONH); ^13^C NMR ((D_6_)DMSO, 75 MHz): *δ* = 110.25 (thiazole C-5), 117.31 (Ar C-3, Ar C-5), 124.13 (Ar C-1), 127.56 (Ar’ C-3, Ar’ C-5), 130.82 (Ar’ C-2, Ar’ C-6), 131.51 (Ar C-2, Ar C-6), 132.28 (Ar’ C-4), 133.92 (Ar’ C-1), 140.71 (thiazole C-4), 156.39 (C=N), 158.43 (C=O), 160.82 (Ar C-4), 163.21 ppm (thiazole C-2); Anal. for C_17_H_13_N_5_O_4_S: calcd: C 53.26, H 3.42, N 18.27, found: C 53.29, H 3.47, N 18.23.

*1-(Bis(4-chlorophenyl)methylene)-4-(5-nitrothiazol-2-yl)semicarbazide****19****:* IR (KBr): ʋ = 3468.13, 3215.44 (N–H str), 2997.48 (aromatic C–H str), 1654.98 (C=O str), 1587.47 (C=N str), 1485.24, 1398.44 (NO_2_ str), 1286.56 (C–N str), 754.19 (C–Cl str); ^1^H NMR ((D_6_)DMSO, D_2_O exchange, 300 MHz): *δ* = 7.39 (d, *J* = 8.7 Hz, 4H, Ar C-3, Ar C-5, Ar’ C-3, Ar’ C-5), 8.07 (d, *J* = 7.5 Hz, 4H, Ar C-2, Ar C-6, Ar’ C-2, Ar’ C-6), 8.60 (s, 1H, thiazole C–H), 9.72 (s, 1H, NH), 10.24 ppm (s, 1H, CONH; ^13^C NMR ((D_6_)DMSO, 75 MHz): *δ* = 109.85 (thiazole C-5), 122.65 (Ar C-3, Ar C-5, Ar’ C-3, Ar’ C-5), 128.26 (Ar C-2, Ar C-6, Ar’ C-2, Ar’ C-6), 129.54 (Ar C-1, Ar’ C-1), 129.81 (Ar C-4, Ar’ C-4), 142.15 (thiazole C-4), 156.29 (C=N), 159.93 (C=O), 164.77 ppm (thiazole C-2); Anal. for C_17_H_11_Cl_2_N_5_O_3_S: calcd: C 46.80, H 2.54, N 16.05, found: C 46.88, H 2.58, N 16.01.

*4-(5-Nitrothiazol-2-yl)-1-(2-oxoindolin-3-ylidene)semicarbazide****20****:* IR (KBr): ʋ = 3431.48, 3335.03, 3219.30 (N–H str), 3132.20 (aromatic C–H str), 1692.69 (isatinyl C=O str), 1652.19 (C=O str), 1600.97 (C=N str), 1464.02, 1338.64 (NO_2_ str), 1234.48 (C–N str); ^1^H NMR ((D_6_)DMSO, D_2_O exchange, 300 MHz): *δ* = 6.92 (dd, *J* = 8.1, 7.1 Hz, 1H, isatinyl C-5), 6.97 (d, *J* = 8.1 Hz, 1H, isatinyl C-7), 7.32 (d, *J* = 7.5 Hz, 1H, isatinyl C-4), 7.38 (dd, *J* = 8.7, 7.6 Hz, 1H, isatinyl C-6), 8.13 (s, 1H, thiazole C–H), 9.05 (s, 1H, NH), 9.77 (s, 1H, isatinyl N–H), 10.86 ppm (s, 1H, CONH); ^13^C NMR ((D_6_)DMSO, 75 MHz): *δ* = 109.92 (thiazole C-5), 128.15 (isatinyl C-3a), 129.24 (isatinyl C-7), 132.58 (isatinyl C-5), 133.30 (isatinyl C-4), 134.38 (isatinyl C-6), 144.06 (thiazole C-4), 144.73 (C=N), 145.17 (isatinyl C-7a), 154.45 (C=O), 160.52 (thiazole C-2), 168.96 ppm (isatinyl C=O); MS: *m/z* = 333.10 (M + 1)^+^; Anal. for C_12_H_8_N_6_O_4_S: calcd: C 43.37, H 2.43, N 25.29, found: C 43.32, H 2.46, N 25.37.

*1-(5-Bromo-2-oxoindolin-3-ylidene)-4–(5-nitrothiazol-2-yl)semicarbazide****21****:* IR (KBr): ʋ = 3433.41, 3302.24, 3144.07 (N–H str), 2914.54 (aromatic C–H str), 1699.34 (isatinyl C=O str), 1668.48 (C=O str), 1577.82 (C=N str), 1554.68, 1408.08 (NO_2_ str), 1238.34 (C–N str); 623.93 (C–Br str); ^1^H NMR ((D_6_)DMSO, D_2_O exchange, 300 MHz): *δ* = 7.08 (d, *J* = 8.7 Hz, 1H, isatinyl C-6), 7.40 (d, *J* = 9.3 Hz, 1H, isatinyl C-7), 7.88 (s, 1H, isatinyl C-4), 8.36 (s, 1H, isatinyl N–H), 8.58 (s, 1H, thiazole C–H), 9.07 (s, 1H, NH), 10.19 ppm (s, 1H, CONH); ^13^C NMR ((D_6_)DMSO, 75 MHz): *δ* = 107.63 (thiazole C-5), 121.94 (isatinyl C-5), 128.73 (isatinyl C-3a), 131.43 (isatinyl C-7), 131.55 (isatinyl C-4), 135.33 (isatinyl C-6), 137.78 (isatinyl C-7a), 140.66 (C=N), 143.59 (thiazole C-4), 158.55 (C=O), 160.81 (thiazole C-2), 171.74 ppm (isatinyl C=O); Anal. for C_12_H_7_BrN_6_O_4_S: calcd: C 35.05, H 1.72, N 20.44, found: C 35.11, H 1.68, N 20.49.

### *In vitro* bioassays

#### Materials

Serotonin (5-HT) for MAO-A assay, benzylamine for MAO-B assay, acetylthiocholine iodide (ACTI) and 5,5'-dithiobis-(2-nitrobenzoic acid) (DTNB) for AChE assay, equine serum butyrylcholinesterase enzyme and butyrylthiocholine iodide (BuTI) for BuChE assay; the reference inhibitors clorgyline hydrochloride (MAO-A), selegiline hydrochloride (MAO-B), donepezil hydrochloride, tacrine hydrochloride (AChE) and phenytoin were purchased from Sigma-Aldrich. Tris-HCl, sodium phosphate and ascorbic acid were obtained from Merck and SD Fine.

#### Animal ethical approval

Ethics Committee of Laboratory Animals in Banaras Hindu University, Varanasi, approved the animal experimentation. Albino Wistar rats weighing between 200 and 220 g were obtained from Central Animal House, Institute of Medical Sciences, Banaras Hindu University (Registration No. Dean/12-13/CAEC/23).

### MAO inhibitory activity

#### Isolation of rat brain mitochondria

Rat brain mitochondria were used as a source of MAO isoforms. All operations were carried out at 4 °C^50^. Male and female adult Wistar rats weighing 200–220 g were decapitated. All brains were rapidly removed and homogenised with a Potter–Elvehjem homogeniser in cold 0.32 M sucrose and 50 mM Tris-HCl, pH 8.2 (10:1, v/w). The homogenate was centrifuged twice at 1000 *g* for 5 min at 4 °C. The resulting supernatant was centrifuged at 20,000 *g* for 20 min. The mitochondrial pellet obtained was suspended in 100 mM sodium phosphate buffer, pH 7.4 (4:1, v/w), fractionated in plastic vials to 500 µl samples and stored at −80 °C. Before use, mitochondria were diluted with 100 mM sodium phosphate buffer to give a working solution of 0.80 mg of protein per millilitre.

### *In vitro* MAO inhibition assay

The experiments were carried out under all the suitable laboratory conditions. The final compounds(**4**–**21**) were evaluated for their *in vitro* MAO-A/-B inhibitory activity in accordance to our previously reported protocol^50^. An aliquot was made to contain mixture of 55 µl mitochondrial suspension (0.80 mg of protein/ml), 90 µl 50 mM Tris-HCl buffer, pH 8.2 and 30 µl solubilising solution (control or inhibitor solution at five different concentrations). The reaction was initiated by adding 25 µl of serotonin (4 mM, substrate for MAO-A) and 25 µl benzylamine (0.1 M, substrate for MAO-B) for the determination of MAO-A and MAO-B activity, respectively. The mixture was incubated at 37 °C for 30 min and the reaction was stopped by the addition of 50 µl of 1 N HCl. The absorbance associated with the production of 5-hydroxyindolacetic acid was measured at 280 nm for the estimation of MAO-A while for MAO-B estimation, the absorbance was measured at 250 nm due to the formation of benzaldehyde. All the assays were performed in triplicate. Control experiments were carried out without inhibitor and blanks were run without mitochondrial suspension.

### Statistical analysis

Calculation of IC_50_ values were determined using GraphPad Prism software (version 5.0) with 95% confidence limits from the plots of inhibition percentages (calculated in relation to a sample of the enzyme treated under the same conditions without inhibitors) versus the logarithm of the inhibitor concentration. Data are expressed as mean ± standard error mean (SEM) of experiments performed in triplicate.

### Reversibility and irreversibility studies for MAO inhibition

Time-dependant inhibition study was performed with the most active inhibitors, compound **21** for MAO-A and compound **4** for MAO-B in order to investigate the reversibility of the observed enzyme using our previously reported method^51^. Compounds **21** and **4** were pre-incubated with the mitochondrial working solution (0.80 mg of protein/ml) for various periods of time viz. 0, 15, 30 and 60 min at 37 °C in Tris-HCl buffer (50 mM, pH 8.2). For this purpose, the concentration of compounds **21** and **4** was equal to 2-fold the measured IC_50_ value for the inhibition of MAO-A (115 µM) and MAO-B (0.424 µM). The reactions were subsequently diluted 2-fold by the addition of 4 mM serotonin and 0.1 M benzylamine to yield a final enzyme concentration of 0.40 mg/ml and the concentrations of the compounds **21** and **4** that are equal to the IC_50_ values. The reactions were further incubated at 37 °C for a further 15 min and the residual enzyme activities were measured and the bar graphs were constructed. All measurements were carried out in triplicate and are expressed as mean ± SEM.

### Cholinesterase inhibitory activity

#### *In vitro* screening of AChE/BuChE activity

The inhibitory potency against rat brain AChE was investigated by the slightly modified colorimetric method of Ellman et al.^52^ using acetylcholine iodide (ACTI) as a substrate and 5,5′-Dithiobis(2-nitrobenzoic acid) (DTNB). Rat brain was used as a source of AChE enzyme. Rats were separately decapitated, the brain cortices quickly removed, weighed and homogenised in cold 10 mM Tris-HCl buffer, pH 7.2, containing 160 mM sucrose. The homogenates were centrifuged at 10,000 *g* for 10 min at 4 °C, and the resulting clear supernatants obtained were used as enzyme sources that were divided into aliquots and stored at −20 °C.

The final compounds (**4**–**21**) were evaluated for their *in vitro* AChE inhibitory activity in accordance to our previously reported protocol^50^. About 30 µl of enzyme sample (AChE) was added to 60 µl of 20 mM sodium phosphate buffer (pH 7.4) and were incubated in the presence of 30 µl of 10 mM DTNB solution with different concentrations of the test compounds. The enzyme reaction was initiated by the addition of 30 µl of 0.8 mM ACTI. The mixtures were incubated for 15 min at 25 °C. The formation of enzymatic product was determined the measurement of absorbance at 415 nm, with 96-well microplate reader (Bio-Tek, ELx800 TM, Instruments Inc., Winooski, VT). Control experiments were carried out without inhibitor, and blanks were run without AChE enzyme. All the assays were performed in triplicate. IC_50_ values were determined using the GraphPad Prism 5.0 (San Diego, CA) and the results were expressed as mean ± SEM.

The BuChE activity was assayed as per the procedure followed for AChE activity except that equine serum BuChE was used as enzyme and butyrylthiocholine iodide (BuTI) was used as substrate.

### Reversibility and irreversibility studies for AChE/BuChE inhibition

To examine whether the observed enzyme inhibition is reversible or irreversible, time-dependant inhibition study^50^ was carried with the lead AChE and BuChE inhibitor compounds, **21** and **17** respectively. Compounds **21** and **17** were pre-incubated with the AChE and BuChE enzymes respectively for various periods of time *viz.* 0, 15, 30 and 60 min at 25 °C in sodium phosphate buffer (20 mM, pH 7.4) and DTNB solution (10 mM). For this purpose, the concentration of compounds **21** and **17** was equal to two-fold the measured IC_50_ value for the inhibition of AChE (0.53 µM) and BuChE (0.048 µM), respectively. The reaction was subsequently diluted 2-fold by the addition of 0.8 mM acetylthiocholine iodide or butyrylthiocholine iodide such that the concentration of the compounds **21** and **17** are equal to the IC_50_ values. The reaction was further incubated at 25 °C for a further 15 min and the residual enzyme activities were measured and the bar graphs were constructed. All measurements were carried out in triplicate and are expressed as mean ± SEM.

### Molecular docking studies

A computer based docking methodology was performed to illuminate the probable binding orientation and interactions of the test compounds within the binding pockets of MAO-A, MAO-B, AChE and BuChE. The molecular docking studies were executed on the PC based machines running on Windows 7 (86×) as operating system.

The molecular docking software included MGL tools 1.5.4 based AutoDock 4.2 (www.scripps.edu) which uses Python 2.7 language – Cygwin C:\program (www.cygwin.com) and Python 2.5 (www.python.com). Discovery Studio Visualizer 3.1 (www.accelrys.com) was employed for visualising the docked molecules.

### Receptor data set preparation

The X-ray crystal structures of human MAO-A co-crystallised with harmine (PDB entry: 2Z5X, resolution = 2.2 Å)^40^, human MAO-B co-crystallised with safinamide (PDB entry: 2V5Z, resolution = 1.6 Å)^41^, recombinant human AChE co-crystallised with donepezil (PDB entry: 4EY7, resolution = 2.35 Å)^42^ and human BuChE (PDB entry: 1P0I, resolution = 2.0 Å)^43^ were retrieved from the Brookhaven Protein Data Bank (http://www.rcsb.org/pdb).

Computational studies were carried out on only one subunit of the MAO (both MAO-A and MAO-B) and cholinesterase (both AChE and BuChE) enzymes. For docking studies, initial protein was prepared by removing all water molecules, heteroatoms, co-crystallised solvent and ligand if any using Discovery Studio Visualizer. In case of both the MAO isozymes, the FAD co-factor was considered in the docking experiments because of its well-known role into the MAO inhibition. This was then loaded on AutoDock Tools (ADT; version 1.5.4). Proper bonds and bond orders were assigned and the missing hydrogen atoms were added. Partial charges for proteins were added using Gasteiger–Marsili charges and Kollman charges, non-polar hydrogen were merged and rotatable bonds were assigned. The file was then saved to pdbqt format^50^^,^^51^.

### Ligand data set preparation

The structures of the test ligands **4**–**21** and reference ligand molecules (harmine for MAO-A, safinamide for MAO-B, donepezil for AChE and tacrine for BuChE) were drawn using the MarvinSketch 5.6 module of Chemaxon tools^53^ and optimised using “Prepare Ligands” in the AutoDock 4.2 and saved in PDB format. The structures of the ligands were then loaded in ADT Tools and flexible torsions were assigned with AutoTors module, and the acyclic dihedral angles were allowed to rotate freely. The file was then converted to pdbqt file format^50^^,^^51^.

### Docking methodology

The docking methodology was performed using the refined protein molecules (2Z5X for MAO-A, 2V5Z for MAO-B, 4EY7 for AChE and 1P0I for BuChE) according to the previously reported protocol^50^^,^^51^.

### *In vitro* DPPH radical scavenging assay

Antioxidant activity of the synthesised compounds (10 mg/ml) was evaluated using the radical scavenging capability against the stable free radical, 2,2′-diphenyl-1-picryl hydrazyl (DPPH**·**). The DPPH**·** scavenging capability was determined using UV based spectrophotometric assay^54^. About 200 µl of the test sample solution (100 µg/ml) was added to 4 ml of 100 µM methanolic DPPH. The sample tubes were wrapped with aluminum foil and kept in dark for 30 min at room temperature and the absorbance was measured at 517 nm using UV–Vis spectrophotometer with solvent and DPPH as blank. Ascorbic acid (100 µg/ml) was used as reference. The percentage inhibition of DPPH activity was estimated using the formula:

Percent (%) inhibition of DPPH activity = Acontrol-AsampleAcontrol×100

where *A*_control_ is the absorbance of DPPH**·** in methanol without an antioxidant and *A*_sample_ is the absorbance of DPPH**·** in the presence of an antioxidant.

### Neurotoxicity screening studies

The neurotoxicity screening was undertaken according to the method followed by the National Institute of Health, using their reported procedures^55^^,^^56^. Male albino mice (CF-1 strain or Swiss, 18–25 g) were used as experimental animals. The animals were housed in metabolic cages, and allowed free access to food and water. The test compounds were suspended in 0.5% methylcellulose/water mixture or in polyethylene glycol (PEG 200).

Minimal motor impairment was measured in mice by the rotarod test. The mice were trained to stay on an accelerating rotarod that rotates at six revolutions per minute. The rod diameter was 3.2 cm. Trained animals were given i.p. injection of the selected inhibitors at a dose of 30 mg/kg. Neurotoxicity was indicated by the inability of the animal to maintain equilibrium on the rod for at least 1 min in each of the three trials.

## Results and discussion

### Chemistry

**Scheme 1.**
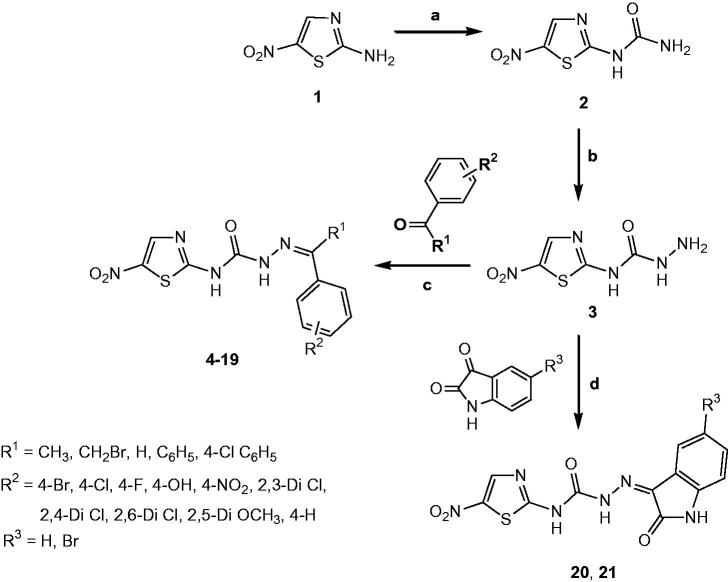
Synthesis of compounds **4**–**21**. Reagents and conditions: (a) Glacial acetic acid/H_2_O, NaOCN, 40–50 °C, 5 h; (b) Ethanol, NH_2_NH_2_·H_2_O, reflux, 18 h; (c) Ethanol, reflux, glacial acetic acid, 35–80 h; (d) Ethanol, reflux, glacial acetic acid, 29–50 h.

The 18 new 2-amino-5-nitrothiazole derived SCZs **4**–**21** presented in this work were prepared through a well-established synthetic route illustrated in Scheme 1 in accordance with the standard reaction conditions^49^. The first intermediate 1-(5-nitrothiazol-2-yl)urea **2** was synthesised in quantitative yield from the commercially available 2-amino-5-nitrothiazole **1** on reaction with sodium cyanate in the presence of glacial acetic acid. The corresponding semicarbazide (**3**) was efficiently prepared by the reaction of **2** with hydrazine hydrate. Finally, the target products 2-amino-5-nitrothiazole derived semicarbazones **4**–**21** were obtained by the acid-catalysed condensation of semicarbazide **3** with appropriate aryl aldehyde or ketone or 5-(un)substituted isatin (Scheme 1).

The physicochemical and spectral data of all synthesised semicarbazones **4**–**21** were in good agreement with their structural composition and are presented in the “Materials and methods” section. Microanalyses (C, H, N) of new compounds agreed with the theoretical value within ±0.4% of the calculated values. The physicochemical characterisation data for the final compounds **4**–**21** are listed in Table 1.

**Table 1.** Physicochemical characterisation data of 2-amino-5-nitrothiazole derived semicarbazones **4**–**21**.


Compound	R^1^	R^2^	R^3^	Mol. Wt. (g mol^−1^)	M.P. (°C)	Yield (%)	cLog*P*^a^	Log*P*^b^	*R*_f_^c^
**4**	CH_3_	4-Br	–	384.21	245–247	59.42	3.08	2.2	0.51
**5**	CH_3_	4-Cl	–	339.76	144–146	65.84	2.91	2.1	0.49
**6**	CH_3_	4-F	–	323.3	267–269	63.92	2.45	1.3	0.47
**7**	CH_3_	4-OH	–	321.31	193–195	25.61	2.01	1.2	0.53
**8**	CH_3_	4-NO_2_	–	350.31	292–294	71.25	2.25	1.4	0.46
**9**	CH_2_Br	4-Br	–	463.1	123–125	38.23	3.80	–	0.52
**10**	H	4-Br	–	370.18	287–289	56.71	3.23	2.5	0.48
**11**	H	4-OH	–	307.29	148–150	49.56	2.16	1.6	0.45
**12**	H	2,3-Di Cl	–	360.18	>300	61.32	3.67	2.6	0.52
**13**	H	2,4-Di Cl	–	360.18	232–234	64.51	3.67	2.5	0.56
**14**	H	2,6-Di Cl	–	360.18	100–102	33.27	3.67	3.1	0.48
**15**	H	2,5-Di OCH_3_	–	351.34	276–278	59.42	2.15	–	0.43
**16**	C_6_H_5_	4-H	–	367.38	Charred at 286	51.54	4.21	3.4	0.41
**17**	C_6_H_5_	4-Cl	–	401.83	182–184	48.86	4.82	3.6	0.44
**18**	C_6_H_5_	4-OH	–	383.38	113–115	29.75	3.91	2.9	0.49
**19**	4-Cl C_6_H_4_	4-Cl	–	436.27	>300	58.67	5.42	3.9	0.54
**20**	–	–	H	332.29	158–160	79.58	1.73	1.1	0.57
**21**	–	–	Br	411.19	297–299	75.46	2.50	1.9	0.49

a MarvinSketch 5.6 generated.

b Determined by standard octanol/water shake flask method.

c Solvent system – CHCl_3_:CH_3_OH:Toluene (7:1:2).

### In vitro bioassays

#### Determination of in vitro MAO inhibitory activity

To achieve a better understanding of the structural requirements for the potency and selectivity towards both the MAO isoforms (MAO-A and MAO-B), all the synthesised semicarbazones **4**–**21** were assessed for their ability to inhibit both the MAO isozymes. Table 2 lists the *in vitro* rat brain MAO-A/B inhibitory activity data of test compounds **4**–**21** as well as the reference compounds clorgyline (for MAO-A) and selegiline (for MAO-B). The inhibitory activities were examined by measuring the effects of each compound on the production of 5-hydroxyindole acetic acid from serotonin (5-HT) for MAO-A and benzaldehyde from benzylamine for MAO-B, using the UV based spectrophotometric MAO enzyme inhibition assay method employing crude rat brain mitochondrial suspension.

**Table 2.** *In vitro* and computational MAO inhibition data for compounds **4**–**21**.

Compound	MAO-A			MAO-B			SI^a^
	*In vitro*	Computational		*In vitro*	Computational		
	IC_50_ (µM) ± SEM	*ΔG* (kcal mol^−1^)	*K*_i_ (µM)	IC_50_ (µM) ± SEM	*ΔG* (kcal mol^−1^)	*K*_i_ (µM)	
**4**	70.19 ± 0.045	−5.93	44.63	0.212 ± 0.004	−6.63	13.84	331.08
**5**	4761 ± 10.83	−5.64	73.85	0.601 ± 0.015	−6.43	19.46	7921.79
**6**	46066 ± 16.72	−5.29	131.54	19.89 ± 0.032	−5.45	100.78	2316.04
**7**	5899 ± 9.84	−5.49	94.27	14.75 ± 0.018	−5.67	70.06	399.93
**8**	2743 ± 3.65	−6.28	25.09	23.88 ± 0.022	−5.55	84.89	114.86
**9**	314 ± 1.89	−5.85	51.78	0.313 ± 0.007	−6.56	15.49	1003.19
**10**	5153 ± 8.82	−5.51	90.88	5.451 ± 0.009	−7.8	1.92	945.33
**11**	11342 ± 11.65	−5.28	134.91	241.5 ± 2.31	−6.75	11.19	46.96
**12**	3369 ± 6.43	−5.74	62.48	8.512 ± 0.132	−7.32	4.3	395.79
**13**	10465 ± 13.92	−5.49	94.68	3.487 ± 0.098	−6.53	16.22	3001.15
**14**	13915 ± 19.68	−5.24	144.64	16.94 ± 0.37	−5.31	127.39	821.43
**15**	55048 ± 24.77	−4.99	220.79	11.13 ± 0.19	−5.85	51.52	4945.91
**16**	89.29 ± 1.26	−6.5	17.11	28.29 ± 0.45	−5.41	109.05	3.15
**17**	4872 ± 5.81	−5.63	74.36	28.52 ± 0.079	−5.5	93.15	170.83
**18**	34305 ± 17.38	−5.25	141.62	157.9 ± 3.71	−5.06	194.35	217.26
**19**	50716 ± 21.75	−5.06	195.81	1.476 ± 0.063	−6.56	15.63	34360.43
**20**	2250 ± 12.81	−5.89	47.83	4.507 ± 0.086	−5.95	43.86	499.22
**21**	57.21 ± 1.36	−5.91	46.69	0.734 ± 0.006	−6.26	25.6	77.94
CLG*	0.0044 ± 0.462	–	–	–	–	–	–
SEL*	67.25 ± 1.02	–	–	0.020 ± 0.0008	–	–	–
HRM*	3.00	−5.3	130.82	7000	–	–	–
SAF*	–	–	–	0.100	−5.93	45.23	–

a SI – The selectivity index is the selectivity for the MAO-B isoform and is given as the ratio of experimental IC_50_(MAO-A)/IC_50_(MAO-B).

* IC_50_ data from Ref. [50].

Reference inhibitors: CLG: Clorgyline; SEL: Selegiline; HRM: Harmine; SAF: Safinamide.

The MAO inhibition data are expressed as IC_50_ values. Based on the MAO screening data (Table 2), it was observed that most of the synthesised compounds exhibited significant inhibitory potential and selectivity towards MAO-B than MAO-A with the IC_50_ values in the micromolar to submicromolar range. The MAO-B selectivity ratios are presented in Table 2.

*Note*: Each IC_50_ value is the mean ± SEM. It refers to the assay concentration of test compound which leads to 50% inhibition of enzyme activity. Level of statistical significance: *p* < .05 versus the corresponding IC_50_ values obtained against MAO-A and MAO-B, as determined by ANOVA/Dunnett’s.

The IC_50_ values toward MAO-A ranged from 57.21 ± 1.36 µM (compound **21**) to 55048 ± 24.77 µM (compound **15**) while for MAO-B ranged from 0.212 ± 0.004 µM (compound **4**) to 157.9 ± 3.71 µM (compound **18**).

Among the synthesised compounds, 1-(5-bromo-2-oxoindolin-3-ylidene)-4–(5-nitrothiazol-2-yl)semicarbazide (compound **21**) was found to be most active MAO-A inhibitor with IC_50_ value of 57.21 ± 1.36 µM, whereas the most active MAO-B inhibitor, 4-(5-nitrothiazol-2-yl)-1-(1-phenylethylidene)semicarbazide (compound **4**) exhibited an IC_50_ value of 0.212 ± 0.004 µM with the selectivity index of 331.08 against MAO-B. Interestingly, compound **19** showed excellent selectivity (SI = 34,360.43) towards MAO-B with an IC_50_ value of 1.476 ± 0.063 µM.

With the optimal length of the linker (–NH–CO–NH–N = C<) in hand, focused structural variations were attempted at the amino and carbimino terminals of the semicarbazone template to explore the structure–activity relationship (SAR) and structure–selectivity relationship (SSR). In general, variations in the lipophilicity caused by the incorporation of monoaryl (compounds **4**–**15**) or diaryl rings (compounds **16**–**19**) or an isatin residue (compounds **20**, **21**) at the carbimino terminal resulted in increased inhibition and selectivity towards MAO-B compared to MAO-A.

Surprisingly, all compounds were active at IC_50_ > 100 µM against MAO-A except **4**, **16**, and **21** (active at IC_50_ < 100 µM). This suggested that replacement of 6-nitrobenzothiazole moiety with 5-nitrothiazole caused a drastic decrease in MAO-A inhibitory activity. This indicated that size of the aryl binding site A seemed to be essential for MAO-A inhibition, but not for MAO-B inhibition.

Very poor MAO-A inhibitory activity was obtained for compounds **6**, **11**, **13**, **14**, **15**, **18**, and **19**. Compound **15** bearing bulky methoxy groups were found to be least active MAO-A inhibitor among all the listed semicarbazones. Hence, we have focused primarily on the MAO-B inhibitory activity exhibited by the compounds for SAR studies.

### SAR for MAO-B inhibition

- Introduction of bromo group at the carbimino terminal phenyl ring increased the potency towards MAO-B (compounds **4**, **9**, and **10**).
- While slight reduction in the activity was observed with chloro substitution (compounds **5**, **12**–**14**, **17**, and **19**) at the carbimino terminal phenyl ring.
- Monochloro-substituted derivative (compound **5**) expressed better MAO-B activity than the dichloro-substituted derivatives (compounds **12**–**14**).
- Substitution with fluoro group (compound **6**) also lead to less active compound.
- Introduction of bulky groups like nitro (compound **8**) and dimethoxy (compound **15**) on the carbimino terminal phenyl ring also resulted in the decrease in the activity towards MAO-B.
- Remarkable reduction in the MAO-B inhibitory activity was observed with 4-hydroxyphenyl substituted derivatives (compounds **7**, **11**, and **18**) at the carbimino terminal.
- Among the hydroxyl substituted analogues (compounds **7**, **11**, and **18**), improvement in MAO-B inhibitory activity was observed by substituting the H atom with a methyl group at R^1^ (compare compounds **7** and **11**). Compound **7** exhibited ∼16.37-fold greater potency relative to compound **11**. While the diaryl substitution (compound **18**) resulted in less active analogue (∼10.7-fold less active relative to compound **7**).
- Among the isomeric dichloro-substituted derivatives (compounds **12**–**14**), 2,4-Dichloro analogue (compound **13**) was found to be more active than 2,3-(compound **12**, ∼2.4-fold less active) and 2,6-Dichloro (compound **14**, ∼4.85-fold less active) substituted analogues. The order of ranking of these inhibitors was **13** (2,4-Dichloro) > **12** (2,3-Dichloro) > **14** (2,6-Dichloro).
- Among the diaryl substituted analogues, the dichloro substitution increased the activity (compound **19**) by ∼19.32-fold relative to monochloro-substituted analogue (compound **17**) with the highest SI (SI = 34360.43 towards MAO-B) while the hydroxyl substitution (compound **18**) resulted in substantial ∼107-fold reduction in the activity.
- Among the isatin-3-substituted semicarbazones, 5-bromo derivative (compound **21**) expressed better MAO-B activity than the unsubstituted derivative (compound **20**).

These results evidenced the influence of the size of hydrophobic moiety at the amino terminal of SCZ template (site A) on the MAO-B inhibitory profile. Notably, decrease in the size of the aryl binding site by replacing 6-nitrobenzothiazole moiety with 5-nitrothiazole moiety at the amino terminal of SCZ template (site A) caused a substantial decrease in the activity toward both MAO enzymes particularly MAO-A. However, increased MAO-B selectivity was observed for all compounds bearing 5-nitrothiazole moiety.

### Kinetic studies of lead MAO inhibitors 21 and 4

**Figure 3.**
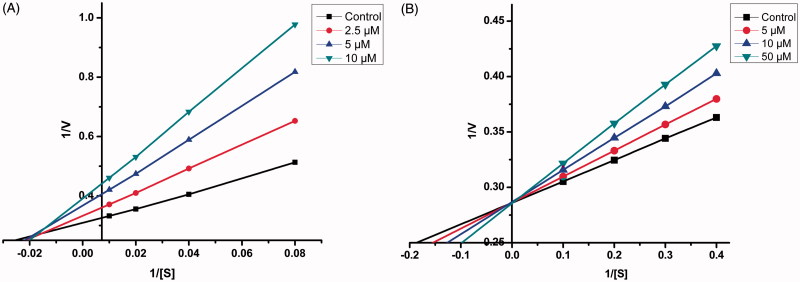
Kinetics of rat brain MAO-A inhibition by compound **21** and MAO-B inhibition by compound **4**. (A) Lineweaver–Burk plot of the rat brain MAO-A catalysed oxidation of serotonin in the absence (control) and presence of various concentrations of compound **21** (2.5, 5, and 10 µM). (B) Lineweaver–Burk plot of the rat brain MAO-B catalysed oxidation of benzylamine in the absence (control) and presence of various concentrations of compound **4** (5, 10, and 50 µM). The rates (V) are expressed as nmol product formed/min/mg protein.

Sets of Lineweaver–Burk plots were constructed to further examine the modes of inhibition of MAO-A enzyme by compound **21** and MAO-B by compound **4**, the representative lead MAO-A and MAO-B inhibitor respectively (Figure 3)^57^. Assessment of Lineweaver–Burk plots indicated that the plot for compound **21** was linear and intersected at the *X*-axis (*K*_m_ remains unaffected while *V*_max_ decreases) while that for compound **4** was linear and intersected at the *Y*-axis (*K*_m_ increases while *V*_max_ remains unaffected). Thus, the pattern indicated that compound **21** inhibited MAO-A non-competitively while compound **4** inhibited MAO-B competitively.

### Determination of K_i_ for lead MAO inhibitors 21 and 4

The dissociation constant (*K*_i_) was determined for the lead MAO-A and MAO-B inhibitors for each mode of inhibition using the GraphPad Prism software revealing the strength of interactions between the enzyme and the inhibitor. *K*_i_ values for non-competitive inhibitor **21** was estimated to be 17.30 ± 1.79 µM against MAO-A while the competitive inhibitor **4** exhibited *K*_i_ value of to be 11.69 ± 0.58 µM against MAO-B.

### Reversibility studies for lead MAO inhibitors 21 and 4

**Figure 4.**
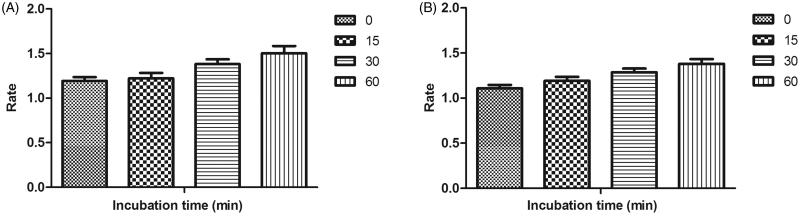
Time-dependant inhibition of (A) MAO-A catalysed oxidation of serotonin by compound **21** (B) MAO-B catalysed oxidation of benzylamine by compound **4**. Rate data are expressed as nmol product formed/min/mg protein.

Time-dependent inhibition studies were performed on the most active MAO-A and MAO-B inhibitors, compound **21** and compound **4** respectively to inspect whether the observed enzyme inhibition was reversible or irreversible. Reversibility test was performed using our previously reported protocol^51^. No time-dependent reduction in the rates of MAO-A catalysed oxidation of serotonin and MAO-B catalysed oxidation of benzylamine respectively was observed when compounds **21** and **4** were pre-incubated with the MAO-A and MAO-B enzymes, respectively, for various periods of time (0, 15, 30, and 60 min). Thus, the above findings speculated that the inhibition of both MAO-A and MAO-B was reversible, at least for the above time period (60 min). Fascinatingly, a slight enhancement of catalytic rates of MAO-A and MAO-B was noticed with increased pre-incubation time of **21** and **4** with the respective enzymes (Figure 4).

### Molecular docking studies of MAO inhibitors

In order to explore the nature of ligand–receptor interactions, molecular docking experiments were performed within the active site of hMAO-A and hMAO-B isoforms using an automated docking program AutoDock 4.2^58^. The co-crystallised structures of hMAO-A (PDB code: 2Z5X)^40^ and hMAO-B (PDB code: 2V5Z)^41^ was used to dock the synthesised compounds. The conformers acquiring the top score among the largest cluster were considered for further structural and binding interaction studies.

MAO-A inhibition results were found to vary dramatically and exhibited poor correlation. However, a satisfactory correlation was observed between the experimental and computational MAO-B inhibition data. The outcomes of the docking studies were theoretically stated in terms of inhibition constants (*K*_i_ values) and free binding energies (*ΔG*) for each of the virtual ligand–receptor complex and are presented in Table 2.

### Pose analysis of MAO-a inhibitors

**Figure 5.**
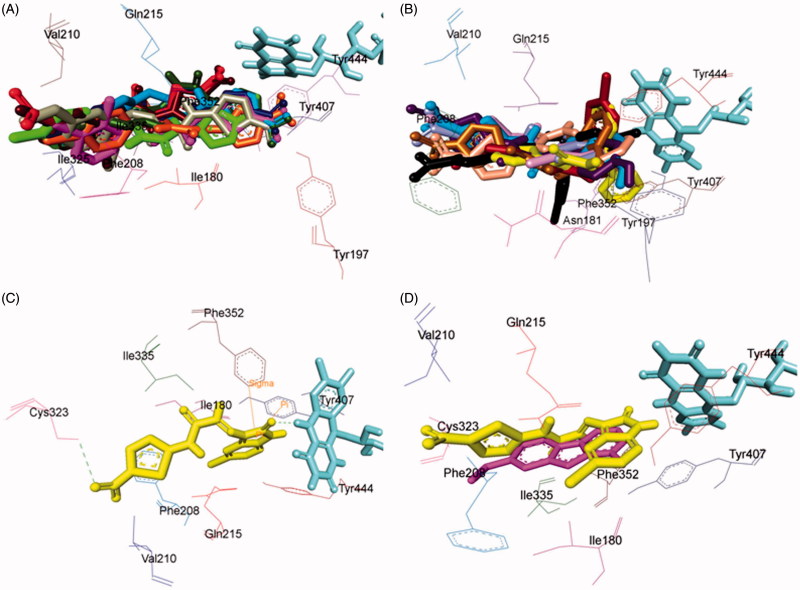
Superimposed MAO-A inhibitors docked into the binding pocket of MAO-A. FAD is displayed in cyan. Selected MAO-A residues are labelled in black. (A) Shared binding orientation of compounds **4**, **5**, **6**, **8**, **9**, **10**, **12**, **13**, and **15** are displayed in dark blue, dark brown, bright green, red, dark orange, light blue, dark pink, dark green, and grey, respectively. (B) Shared binding orientation of compounds **7**, **11**, **14**, **16**, **17**, **18**, **19**, **20**, and **21** are displayed in dark purple, light blue, light brown, yellow, maroon, light orange, black, light purple, and light pink, respectively. (C) Binding orientation of compound **21** (yellow) within the MAO-A binding pocket showing π–π (orange lines) and hydrogen bonding (green dashed lines) interactions. (D) Superimposed binding orientation of compound **21** (yellow) within the MAO-A binding pocket originally docked with harmine (dark pink).

Assessment of the virtual ligand–receptor complexes of all compounds within the catalytic site of MAO-A resulted in the below mentioned interpretations: on the whole, all the test compounds occupied the active site of MAO-A and were framed within the binding pocket constituted by the residues Ile180, Asn181, Tyr197, Phe208, Val210, Gln215, Cys323, Ile 325, Ile335, Phe352, Tyr407, Tyr444, and FAD which was analogous to the reference MAO-A inhibitor, harmine (Figure 5(A) and (B))^40^.

Compounds **4**, **5**, **6**, **8**, **9**, **10**, **12**, **13**, and **15** (Figure 5(A)) shared a common binding orientation within the active site of MAO-A in which the 5-nitrothiazole nucleus occupied the centre of the cavity toward FAD while the carbimino terminal of the semicarbazino linker was situated toward the opening of the cavity. Whereas in the compounds **7**, **11**, **14**, **16**, **17**, **18**, **19**, **20**, and **21**, all sharing common binding orientation, the case was reversed, i.e. the carbimino terminal was located towards the FAD and the 5-nitrothiazole moiety extended toward the opening of the cavity space (Figure 5(B)). Residues Tyr407, Tyr444, and FAD formed the bottom of the aromatic cage which accounted to the binding orientation of the compounds and consequently their activity, binding affinity, selectivity, and stability.

All compounds showed one or more hydrogen bonding interactions excluding compounds **6**, **15**, **17**, **18**, and **19**. Additionally, all the compounds showed π–π interactions apart from compounds **11** and **14**. Thus, it can be concluded that the least active MAO-A inhibitors, i.e. compounds **6**, **15**, **18**, and **19** (active at IC_50_ > 100 µM) lack hydrogen bonding interactions which may be the reason for their excessively reduced activity towards MAO-A. Further, in the active compounds *viz.***4**, **16**, and **21** (active at IC_50_ < 100 µM), preferably hydrogen bonding and π–π interactions were observed to be accountable for mediating MAO-A inhibition.

### Binding mode of lead MAO-a inhibitor 21

Examination of one of the best-ranked docking solutions of lead MAO-A inhibitor **21** (Figure 5(C)) (*ΔG* = −5.91 kcal mol^−1^; computational *K*_i_ = 46.69 µM) revealed that semicarbazone linker lies in the centre of the cavity with the 5-bromoisatin moiety facing towards FAD while the 5-nitrothiazole moiety was positioned toward the opening of the MAO-A receptor. The 5-membered ring of isatin was involved in π–π interaction with Tyr407 at an inter-plane distance of ∼4.64 Å. In addition, the molecule was also stabilised by hydrogen bonding interactions between (i) oxygen atom of NO_2_ of 5-nitrothiazole and hydrogen atom of SH of Cys323 and (ii) oxygen atom of C=O of isatin and H5 of FAD. Figure 5(D) illustrated the docked pose of compound **21** (yellow) within the MAO-A binding pocket superimposed with the docked pose of harmine (dark pink) which indicated the similarity in their binding orientation.

### Pose analysis of MAO-B inhibitors

**Figure 6.**
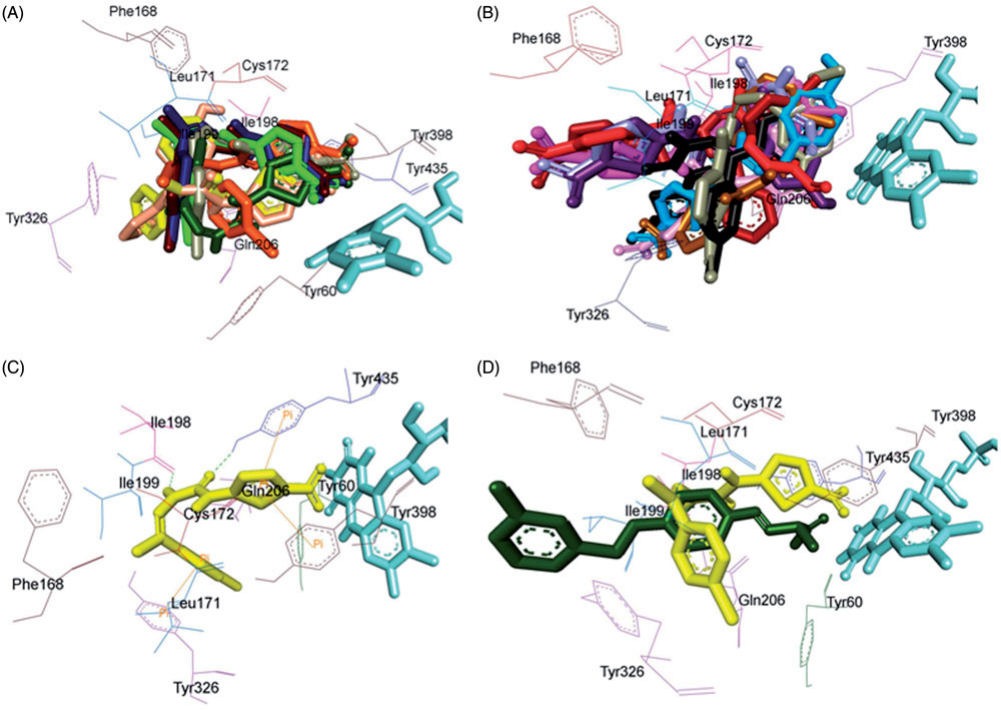
Superimposed MAO-B inhibitors docked into the binding pocket of MAO-B. FAD is displayed in cyan. Selected MAO-B residues are labelled in black. (A) Shared binding orientation of compounds **4**, **5**, **6**, **9**, **10**, **13**, **16**, and **18** are displayed in dark blue, dark brown, bright green, dark orange, grey, dark green, yellow, and light orange, respectively. (B) Shared binding orientation of compounds **7**, **8**, **11**, **12**, **14**, **15**, **17**, **19**, **20**, and **21** are displayed in dark purple, red, light blue, dark pink, light brown, grey, maroon, black, light purple, and light pink, respectively. (C) Binding orientation of compound **4** (yellow) within the MAO-B binding pocket showing π–π (orange lines) and hydrogen bonding (green dashed lines) interactions. (D) Superimposed binding orientation of compound **4** (yellow) within the MAO-B binding pocket originally docked with safinamide (dark green).

All the inhibitors were located within the catalytic binding pocket of MAO-B. The binding mode of all the test compounds within MAO-B traversed both the binding pockets, entrance cavity lined by the residues Leu171, Phe168, Ile198, Ile199, and Tyr326; and substrate cavity bordered by the residues Tyr60, Cys172, Gln206, Tyr398, and Tyr435 which is almost analogous to the binding sites of safinamide, the reference MAO-B inhibitor^41^. Compounds **4**, **5**, **6**, **9**, **10**, **13, 16**, and **18** (Figure 6(A)) shared a common binding orientation within the active site of MAO-B, in which the 5-nitrothiazole nucleus was located in the substrate cavity while the carbimino side chain extended towards the entrance cavity of MAO-B. Whereas the compounds **7**, **8**, **11**, **12**, **14**, **15**, **17**, **19**, **20**, and **21** (Figure 6(B)) all sharing a common binding orientation, the case was exactly reversed, i.e. the carbimino terminal bearing aromatic/heteroaromatic ring was situated towards the FAD and the 5-nitrothiazole moiety flipped towards the entrance cavity space of MAO-B (Figure 6(B)).

All the compounds showed hydrogen bonding interactions except compounds **12** and **13**. Hydrogen bonding interactions with Leu171 was observed for compound **15**; with Cys172 for compounds **17**, **18**, and **20**; with Tyr188 for compound **11**; with Ile198 for compounds **4**, **5**, **6**, **8**, **9**, **10**, and **20**; with Thr201 for compounds **11** and **19**; with Gln206 for compounds **7**, **14**, **19**, and **21**; with residue Tyr326 are observed with compounds **8** and **17**; with Tyr398 for compounds **16** and **18**; with Tyr435 for compounds **4**, **5**, **6**, **9**, and **10**; and with FAD for compounds **7**, **8**, **19**, and **21**. Also, all compounds showed π–π interactions except compounds **6**, **8**, **16**, **17**, and **18**. π–π interactions were observed with Tyr326 for compounds **4**, **5**, and **13**; with Tyr398 for compounds **4**, **7**, **11**, **12**, **13**, **15**, and **19**; with Tyr435 for compounds **4**, **5**, **9**, **10**, **11**, **12**, **13**, **14**, **15**, and **19**. Additionally, π–σ interaction has been observed with Gln206 for compound **14**; with Phe208 for compound **20**; with Tyr435 for compound **21**; and with FAD for compounds **7** and **21**. Thus, in most of the MAO-B active compounds, preferably π–π and hydrogen bonding interactions were observed to be responsible for mediating MAO-B inhibition.

### Binding mode of lead MAO-B inhibitor 4

Visual inspection of one of the best-ranked docking solution of lead MAO-B inhibitor **4** revealed that the entire molecule was stabilised within both the cavities with the rigid hydrophobic 5-nitrothiazole moiety caged into the substrate cavity, while the arylidene ring present at the carbimino terminal extended toward the entrance cavity of the MAO-B. The compound was found to be stabilised by hydrogen bonding and π–π interactions. Hydrogen bonding interactions were observed between oxygen atom of carbimino C=O and hydrogen atom of OH of Tyr435; and hydrogen atom of NH in carbimino linker and oxygen atom of Ile198 (Figure 6(C)). In addition, π–π interactions between the thiazole ring and the phenyl ring of Tyr398 and Tyr435; and carbimino terminal phenyl ring with phenyl ring of Tyr326 (Figure 6(C)). These interactions, overall, resulted in the firmness of the ligand within the MAO-B catalytic site and hence confirmed the stability of the ligand–receptor complex. Figure 6(D) demonstrates the docked pose of compound **4** (yellow) within the catalytic site of MAO-B superimposed with the docked pose of safinamide (dark green). Compound **4** was found to occupy the active site of MAO-B but displayed a different mode in comparison to the reference inhibitor safinamide (dark green).

To provide additional support to our findings, further, we determined global minimum energy conformations for the docking simulations. These calculations were executed onto hMAO-A and hMAO-B receptor models and the recognition of the most active inhibitor **4** was assessed using the interaction energy parameter (Table 3)^51^^,^^59^.

**Table 3.** Interaction energies of compound **4** within the binding pockets of hMAO-A and hMAO-B.

Compound	Enzyme	*Δ*E_int_ (kcal mol^−1^)^a^	vdW (kcal mol^−1^)^b^	EI (kcal mol^−1^)^c^
**4**	hMAO-A	−7.00	−7.43	0.3
	hMAO-B	−7.81	−7.82	0.1

a Total interaction energy.

b van der Waals contribution.

c Electrostatic interaction.

Compound **4** highlighted a better interaction to hMAO-B than hMAO-A enzymes (Table 3) and it is in agreement with the experimental data as reported in Table 2. The interaction energy components analysis indicated a complete preference of compound **4** with respect to the B isoform in terms of both vdW and electrostatic contributions.

### *In vitro* AChE inhibition assay

All the synthesised compounds **4**–**21** were subjected to Ellman’s test in order to evaluate their potency to inhibit the rat brain AChE by the colorimetric method^52^. Donepezil and tacrine were used as reference standards. Selected compounds viz. **4**, **5**, **16**, **17**, **19**, and **21** were further investigated for their ability to inhibit equine serum BuChE. The inhibitory data are presented in terms of IC_50_ values in Table 4.

**Table 4.** *In vitro* and computational cholinesterase inhibition data for compounds **4–21.**

Compound	AChE			BuChE		
	*In vitro*	Computational		*In vitro*	Computational	
	IC_50_ (µM) ± SEM	*ΔG* (kcal mol^−1^)	*K*_i_ (µM)	IC_50_ (µM) ± SEM	*ΔG* (kcal mol^−1^)	*K*_i_ (µM)
**4**	0.340 ± 0.007	−6.29	24.54	0.499 ± 0.003	−6.47	18.17
**5**	356.1 ± 4.73	−5.29	132.58	0.553 ± 0.038	−6.21	27.87
**6**	467.9 ± 4.98	−5.05	197.98	NT	−5.15	168.81
**7**	12.69 ± 0.62	−6.16	30.68	NT	−5.37	114.9
**8**	45.53 ± 1.36	−6.21	28.08	NT	−5.31	129.13
**9**	3.926 ± 0.014	−6.51	16.96	NT	−5.98	41.56
**10**	96.37 ± 2.81	−5.6	78.3	NT	−6.23	27.26
**11**	584.4 ± 7.92	−5.03	205.05	NT	−5.22	148.87
**12**	101.2 ± 1.58	−5.63	74.59	NT	−5.91	46.66
**13**	140.7 ± 3.11	−5.46	99.58	NT	−6.3	24.18
**14**	10.67 ± 0.56	−6.08	34.85	NT	−5.69	67.04
**15**	872.0 ± 4.38	−5.4	109.84	NT	−5.22	149.56
**16**	4.864 ± 0.81	−6.68	12.76	1.757 ± 0.035	−6.19	28.79
**17**	11.54 ± 0.35	−6.38	21.08	0.024 ± 0.002	−6.99	7.52
**18**	2.662 ± 0.084	−7.3	4.46	NT	−6.03	38.26
**19**	1.450 ± 0.047	−7.05	6.8	5.718 ± 0.874	−6.22	27.57
**20**	54.85 ± 0.92	−5.77	58.85	NT	−6.44	19.09
**21**	0.2645 ± 0.009	−6.71	11.97	5.368 ± 0.278	−6.33	23.02
**DPZ**	0.021 ± 0.005	−6.01	39.45	–	–	–
**TAC**	0.225 ± 0.04	–	–	5.1 ± 0.2*	−5.35	119.26

* Data from Ref. [60] and is expressed in nanomolars (nM).

NT – Not tested.Each IC_50_ value is the mean ± SEM. Level of statistical significance: *p* < .05 versus the corresponding IC_50_ values obtained against AChE, as determined by ANOVA/Dunnett’s. Reference inhibitors: DPZ – Donepezil, TAC – Tacrine.

All the synthesised compounds **4**–**21** were found to show AChE inhibitory activity in micromolar to sub-micromolar range. Among them, compound **21,** 1-(5-bromo-2-oxoindolin-3-ylidene)-4-(5-nitrothiazol-2-yl)semicarbazide (IC_50_ = 0.264 ± 0.009 µM) emerged as the most active inhibitor, followed by compound **4**, (1-(1-(4-bromophenyl)ethylidene)-4-(5-nitrothiazol-2-yl)semicarbazide, IC_50_ = 0.340 ± 0.007 µM). It was noted that both these compounds possessed bromo substituent at the carbimino terminal of the semicarbazone template. Further, the activity of compound **21** was observed to be almost equivalent to the reference inhibitor tacrine.

A thorough examination of the inhibition data presented in Table 4 guided us to eventual structure–activity relationships (SAR).

### SAR of AChE inhibition

- In general, improvement in AChE inhibitory activity was observed by substituting the H atom with a methyl group at R^1^ (compare **4**, **7**, **8**, **9** with **10**, **11**, **12**, **13**, **15**; exception **5**, **6**, and **14**)
- Introduction of bromo group at the carbimino terminal phenyl ring increased the activity (compounds **4** and **9**), while chloro (compound **5**) and fluoro substitution (compound **6**) decreased the activity by several folds (greater than ∼1000-fold).
- Substitution with hydroxyl group at the carbimino terminal phenyl ring at R^2^ along with the methyl group at R^1^ (compound **7**) lead to moderately active compound.
- Among the hydroxyl substituted analogues (compounds **7**, **11**, and **18**), improvement in AChE inhibitory activity was observed by substituting the H atom with a methyl group at R^1^ (compare compounds **7** and **11**). Compound **7** exhibited ∼46-fold greater potency relative to compound **11**. While the diaryl substitution (compound **18**) resulted in more active analogue (∼4.97-fold more active relative to compound **7**).
- Introduction of bulky groups like nitro (compound **8**) resulted in the decrease in the activity and dimethoxy substitution (compound **15**) caused a substantial reduction in the activity.
- Monochloro- and dichloro-substitution reduced the activity. Dichloro-substituted derivatives (compounds **12**–**14**) expressed better AChE activity than the monochloro-substituted derivative (compounds **5**).
- Among the isomeric dichloro-substituted derivatives (compounds **12**–**14**), 2,6-dichloro analogue (compound **14**) was found to be more active than 2,3- (compound **12**, ∼9.4-fold less active) and 2,4-dichloro (compound **14**, ∼13.2-fold less active) substituted analogues. The order of ranking of these inhibitors was **14** (2,6-dichloro) > **12** (2,3-dichloro) > **13** (2,4-dichloro).
- Among the diaryl substituted analogues (**16**–**19**), the dichloro substitution increased the activity (compound **19**) by ∼7.95-fold relative to monochloro-substituted analogue (compound **17**) while the hydroxy substituted (compound **18**) and unsubstituted analogues (compound **16**) were observed to be ∼1.83-fold and ∼3.35-fold less active relative to compound **19**.
- Among the isatin-3-subsituted semicarbazones (**20** and **21**), 5-bromo-substituted derivative (compound **21**) was found to be more active than the unsubstituted derivative (compound **20**).

Thus, the compounds bearing bromo substituent on the carbimino terminal aryl ring/isatin ring displayed better potency against AChE demonstrating the role of electronegative bromo substituent in the stabilisation of the hydrophobic ring within the active site gorge of AChE.

### *In vitro* BuChE inhibition assay

Selected compounds *viz.***4**, **5**, **16**, **17**, **19**, and **21** were further investigated for their ability to inhibit equine BuChE, their IC_50_ values are presented in Table 4. The investigated compounds were found to show BuChE inhibitory activity in micromolar range. Interestingly compound **17**, 1-((4-Chlorophenyl)(phenyl)methylene)-4-(5-nitrothiazol-2-yl)semicarbazide (IC_50_ = 0.024 ± 0.002 µM) emerged as the most active inhibitor followed by compound **4**, (1-(1-(4-bromophenyl)ethylidene)-4-(5-nitrothiazol-2-yl)semicarbazide (IC_50_ = 0.499 ± 0.003 µM). A thorough examination of the inhibition data presented in Table 4 guided us to eventual structure–activity relationships.

### SAR of BuChE inhibition

- Introduction of bromo group at the carbimino terminal phenyl ring (site C) increased the activity (compound **4**), while chloro substitution (compound **5**) slightly decreased the activity. Further, replacement of phenyl ring at the carbimino terminal by heteroaryl ring (compound **21**) enormously reduced the activity, *viz.* by ∼10.75-fold compared to compound **4** and ∼9.7-fold compared to compound **5**.
- Among the tested diaryl substituted analogues (**16**, **17** and **19**), the monochloro-substituted analogue (compound **17**) increased the activity, while dichloro substitution (compound **19**) decreased the activity by ∼238-fold. The unsubstituted analogue (compound **16**) was observed to be ∼73-fold less active relative to compound **17**.
- Among the heteroaryl substituted semicarbazones (**20** and **21**), 5-bromo-substituted derivative (compound **21**) was found to be more active than the unsubstituted derivative (compound **20**).

This clearly indicates the requirement of a hydrophobic aryl binding moiety with optimal electronegativity at the carbimino terminal for effective BuChE inhibition.

### Kinetic study of lead AChE/BuChE inhibitor 21 and 17

**Figure 7.**
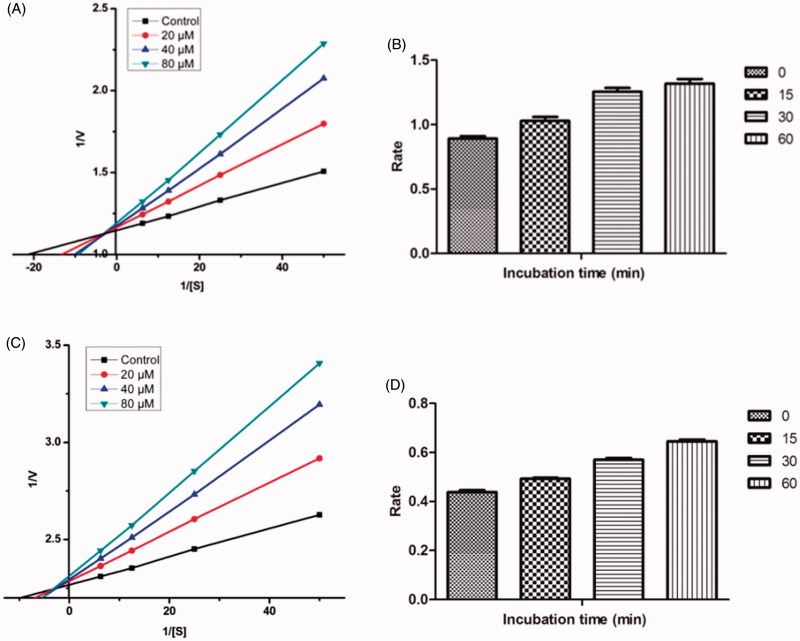
(A) Kinetics of rat brain AChE inhibition by compound **21**. Lineweaver–Burk plot of the rat brain AChE catalysed oxidation of ACTI in the absence (control) and presence of various concentrations of compound **21** (20, 40, and 80 µM). (B) Time-dependant inhibition of AChE catalysed oxidation of ACTI by compound **21**. Rate data are expressed as nmol product formed/min/mg protein. (C) Kinetics of equine serum BuChE inhibition by compound **17**. Lineweaver–Burk plot of the equine serum BuChE catalysed oxidation of BuTI in the absence (control) and presence of various concentrations of compound **17** (20, 40, and 80 µM). (D) Time-dependant inhibition of BuChE catalysed oxidation of BuTI by compound **17**. Rate data are expressed as nmol product formed/min/mg protein.

In order to enlighten the mechanism of action of this family of compounds on AChE and BuChE, kinetic studies were performed with the most promising compounds, **21** and **17**, using rat brain AChE and equine serum BuChE, respectively. Graphical analysis of the reciprocal Lineweaver–Burk plots (Figure 7(A,C)) demonstrated increased slopes (decreased *V*_max_) and intercepts (higher *K*_m_) at increasing concentration of the inhibitors signifying a mixed-type inhibition and consequently supports the dual site binding of these compounds.

### Reversibility studies of lead AChE/BuChE inhibitor 21 and 17

Time-dependent inhibition study of the most active AChE and BuChE inhibitor, compounds **21** and **17**, was done to explore whether the observed enzyme inhibition is reversible or irreversible. There is no time-dependent reduction in the rates of AChE/BuChE catalysed oxidation of ACTI/BuTI when compounds **21** and **17** were pre-incubated with the AChE/BuChE for various periods of time i.e. 0, 15, 30, and 60 min (Figure 7(B,D)). From this result, it may be deduced that the inhibition of AChE/BuChE is reversible, at least for the time period (60 min). An increase of AChE/BuChE catalytic rates was observed with the increase in the pre-incubation time of **21** and **17** with the enzyme.

### Molecular docking studies of AChE and BuChE inhibitors

To better understand the molecular mechanism of recombinant human AChE (rhAChE) and human BuChE inhibition, docking simulations were performed for all the test compounds. The test compounds were docked into the binding pocket of rhAChE/BuChE in order to estimate the ligand binding affinity by means of AutoDock 4.2 using X-ray model of the rhAChE with the PDB code 4EY7 co-crystallised with donepezil and BuChE with PDB code 1P0I obtained from the Protein Data Bank, respectively^42–44^. For each compound, the corresponding theoretical inhibition constants (*K*_i_ values) and estimated free energies of binding (*ΔG*) for each virtual enzyme–ligand complex has been considered, and in accordance to the biological data, confirmed the better accommodation into the active site of rhAChE and BuChE (Table 4).

### Pose analysis of AChE inhibitors

**Figure 8.**
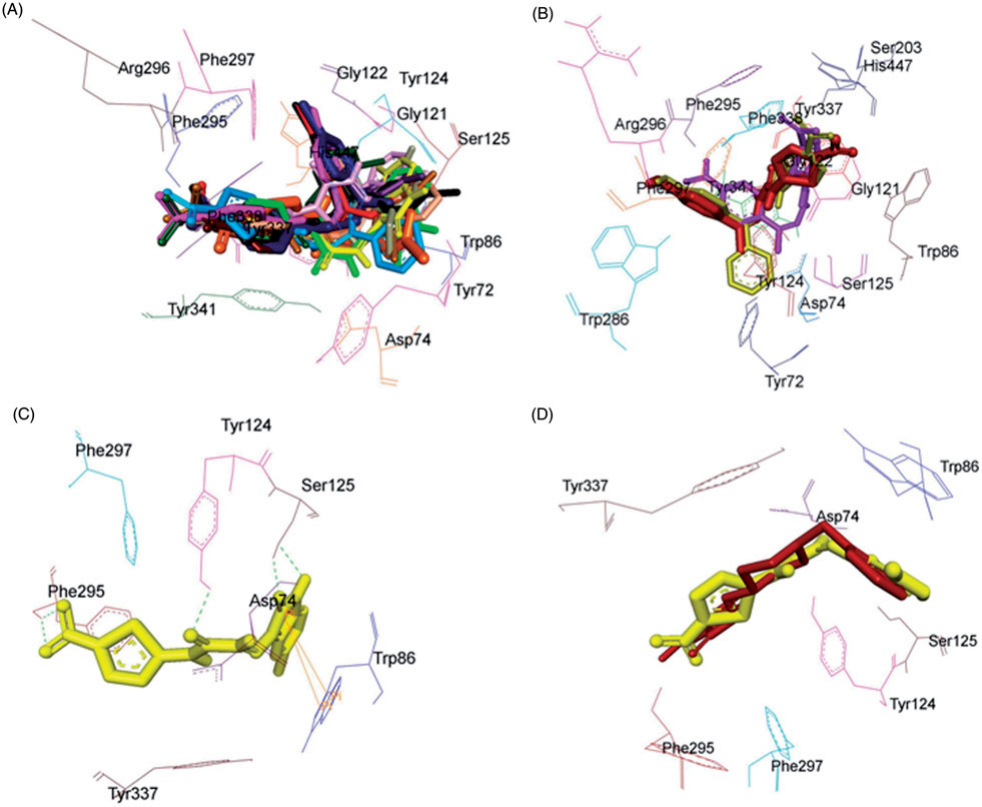
Superimposed AChE inhibitors docked into the binding pocket of AChE. Selected AChE residues are labeled in black. (A) Shared binding orientation of compounds **4–6**, **9–17**, and **19**–**21** are displayed in dark blue, dark brown, dark purple, red, orange, dark pink, dark green, light brown, light pink, grey, light orange, light blue, black, bright green, and yellow, respectively. (B) Shared binding orientation of compounds **7**, **8**, and **18** are displayed in maroon, light purple, and yellowish green, respectively. (C) Binding orientation of compound **21** (yellow) within the AChE binding pocket showing hydrogen bonding (green dashed lines) and π–π (orange lines) interactions. (D) Superimposed binding orientation of compound **21** (yellow) within the AChE binding pocket originally docked with donepezil (maroon).

Investigation of the computationally docked binding pose of all compounds within the rhAChE binding pocket resulted in the subsequent findings: All the test compounds were found to fit into the gorge of rhAChE formed by catalytic anionic site (CAS) and peripheral anionic site (PAS) and were surrounded by the residues Tyr72, Asp74, Trp86, Gly121, Gly122, Tyr124, Ser125, Ser203, Tyr133, Ser203, Trp286, Phe295, Arg296, Phe297, Tyr337, Phe338, Tyr341, and His447 (Figure 8(A,B)) revealing a similar binding orientation as that of reference inhibitor donepezil^42^.

The compounds were found to be stabilised by hydrogen bonding and π–π interactions. All compounds showed one or more hydrogen bonding interactions. Hydrogen bonding interactions were observed with residue Asp74 and Trp86 for compound **20**; likewise with Gly121 and Gly122 for compounds **7**, **8**, and **18**; with Tyr124 for compounds **4**, **7**, **8**, **11**, **16**, **17**, **18**, **19**, and **21**; with Ser125 for compounds **15** and **21**; with Ser203 for compounds **7** and **11**; with Ala204 for compounds **8** and **18**; with Trp286 for compound **5**; with Phe295 for compounds **4**, **5**, **6**, **8**, **9**, **10**, **13**, **14**, **15**, **16**, **17**, **19**, **20**, and **21**; with Arg296 for compounds **7**, **9**, **11**, **12**, **13**, **17**, **18**, and **19**; with Tyr337 for compounds **6** and **10**; and with Tyr341 for compound **8**.

In addition, all the compounds showed π–π interactions except compounds **6**, **7**, **11**, and **15**. π–π interaction with residue Phe297 was observed for compounds **5** and **8**; with Tyr341 for compounds **4**, **5**, **8**, **16**, and **18**; with Trp86 for compounds **9**, **10**, **13**, **14**, **15**, **16**, **20**, and **21**. Moreover, π–cation interaction was observed with His447 for compounds **12** and **18**. Also, π–σ interaction with Trp86 was observed for compound **16**; likewise with Phe297 for compound **17**; and with His447 for compound **18**. From the Table 4, it could be seen that the compounds **6**, **7**, **11**, and **15** were observed to lack π–π interactions which might lead to their lower inhibitory capacity against AChE.

### Binding mode of lead AChE inhibitor 21

Evaluation of the virtual complex between the lead AChE inhibitor **21** and AChE revealed that compound **21** could perfectly fit into the gorge of AChE and simultaneously interact with the CAS and PAS of AChE. The thiazole moiety was observed to bind to the PAS while the 5-bromoisatin moiety occupied the CAS, whereas the linker occupied the middle of the gorge between CAS and PAS (Figure 8(C)). The complex was further stabilised by hydrogen bonding interactions between oxygen atom of carbimino C=O and hydrogen atom of OH of Tyr124; oxygen atom of C=O of isatin and hydrogen atom of OH of Ser125; oxygen atom of NO_2_ of thiazole and hydrogen atom of NH of Phe295; and hydrogen atom of NH of isatin and oxygen atom of OH of Ser125. Further, aromatic ring of isatin was involved in π–π interaction with aromatic ring of Trp86 at an inter-plane distance of ∼5.38 and ∼4.86 Å, respectively. The docking results indicated that compound **21** was a mixed-type inhibitor of AChE, which was consistent with our kinetic analysis result. Superimposition of rhAChE:**21** complex with the rhAChE:donepezil complex suggested that compound **21** shared a similar binding mode of donepezil (Figure 8(D)).

### Pose analysis of BuChE inhibitors

**Figure 9.**
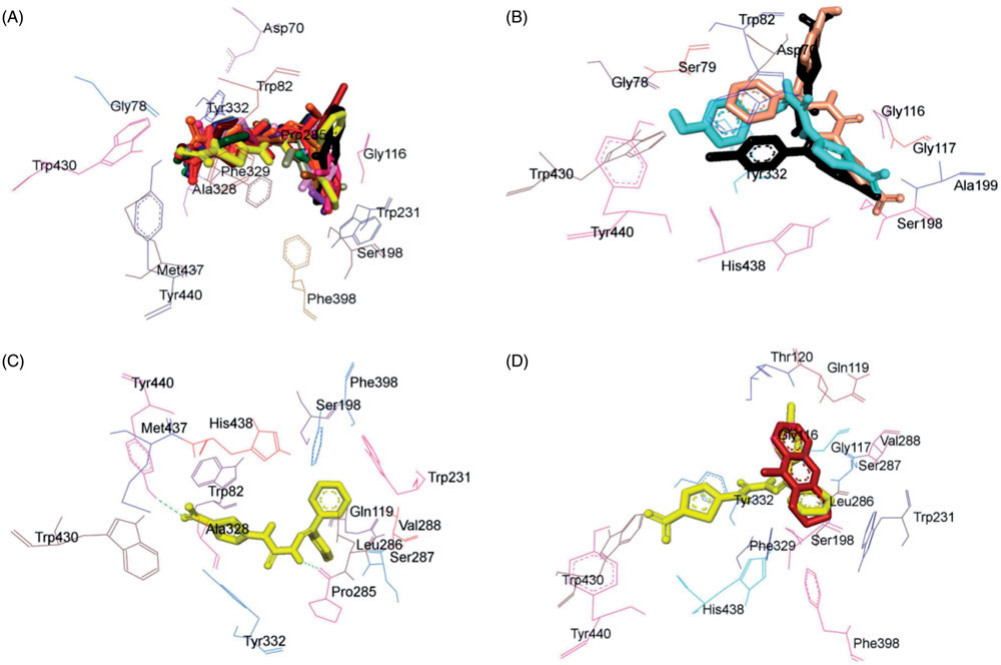
Superimposed BuChE inhibitors docked into the binding pocket of BuChE. Selected BuChE residues are labelled in black. (A) Shared binding orientation of compounds **4**–**10**, **12–17**, and **20–21** are displayed in dark blue, dark brown, bright green, dark purple, red, dark orange, light blue, dark pink, dark green, light brown, grey, yellow, maroon, light purple, and light pink, respectively. (B) Shared binding orientation of compounds **11**, **18**, and **19** are displayed in fluorescent blue, crimson, and black green, respectively. (C) Binding orientation of compound **17** (yellow) within the BuChE binding pocket showing hydrogen bonding (green dashed lines) interactions. (D) Superimposed binding orientation of compound **17** (yellow) within the BuChE binding pocket originally docked with tacrine (maroon).

Investigation of the computationally docked binding pose of all compounds within the BuChE binding pocket resulted in the subsequent findings: all the test compounds were found to fit into the gorge of BuChE formed by catalytic anionic site (CAS) and peripheral anionic site (PAS) and were surrounded by the residues Asp70, Gly78, Ser79, Trp82, Gly116, Gly117, Ser198, Ala199, Pro285, Val288, Leu286, Ser287, Ala328, Phe329, Tyr332, Phe398, Trp430, Met437, Tyr440, and His438 (Figure 9(A,B)) revealing a similar binding orientation as that of reference inhibitor tacrine^43^^,^^44^.

The compounds were found to be stabilised by hydrogen bonding and π–π interactions. All compounds showed one or more hydrogen bonding interactions. Hydrogen bonding interactions were observed with residue Trp82 for compounds **4**–**6**, **8**, and **10**; likewise with Trp430 for compounds **4**, **5**, **6**, **8**, **10**, **11**, **13**, **15**, and **16**; with Tyr440 for compounds **4**, **5**, **6**, **8**, **10**, **11**, **13**, **15**, **16**, **17**, and **21**; with Pro285 for compounds **4**, **5**, **6**, **7**, **9**, **10**, **12**, **13**, **14**, **15**, **17**, **20**, and **21**; with Tyr332 for compounds **8** and **12**; with Gly78, Gly116, Gly117, Ser198, and Ala199 for compound **11**; with Gln67 for compound **18**; with Asp70 for compound **19**; with Leu286 for compound **20**; and Ser287 for compound **21**.

In addition, all the compounds showed π–π interactions except compounds **7**, **8**, **9**, **14**, **17**, and **19**. π–π interaction with residue Trp82 was observed for compounds **11** and **18**; with Tyr332 for compounds **4**, **5**, **10**, **11**, **13**, and **21**. Moreover, π–σ interaction was observed with Trp82 and Trp231 for compounds **6** and **12**, respectively. Also, π–cation interaction with Tyr82 was observed for compound **11**; likewise with Tyr332 for compounds **10**, **11**, **13**, **15**, **20**, and **21**; and with His438 for compound **16**. Lack of π–π interaction for compound **17** indicates this interaction is not essential to exert inhibition against BuChE.

### Binding mode of lead BuChE inhibitor 17

Assessment of the virtual complex between the lead inhibitor **17** and BuChE revealed that compound **17** could perfectly occupy the gorge of BuChE and simultaneously interact with the CAS and PAS of BuChE. The thiazole moiety was observed to bind to the PAS while the diaryl moiety occupied the CAS, whereas the linker occupied the middle of the gorge between CAS and PAS (Figure 9(C)). The complex was further stabilised by two hydrogen bonding interactions; one between hydrogen atom of carbimino NH and oxygen atom of Pro285; and the other between oxygen atom of NO_2_ of thiazole and hydrogen atom of OH of Tyr440. The docking results indicated that compound **17** was a mixed-type inhibitor of BuChE, which was consistent with our kinetic analysis result. Superimposition of BuChE:**17** complex with the BuChE:tacrine complex suggested that compound **17** shared a similar binding mode of tacrine (Figure 9(D)).

### Comparison of the effect of aryl binding site on MAO-B and AChE inhibition

**Figure 10.**
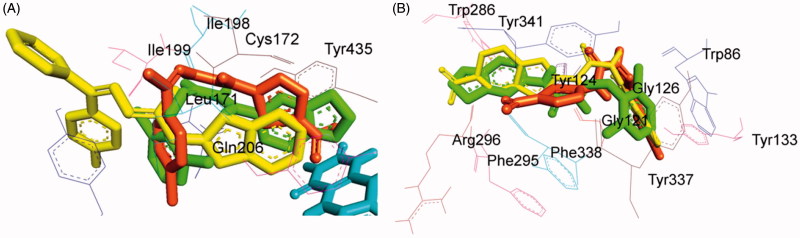
(A) Superimposed binding mode of lead 2-amino-5-nitrothiazole derived semicarbazone (compound **4**, orange) originally docked with our previously reported lead inhibitors (**i**) and (**ii**) (yellow and green, respectively) within the MAO-B active site. (B) Superimposed binding mode of lead 2-amino-5-nitrothiazole derived semicarbazone (compound **21**, orange) originally docked with our previously obtained lead inhibitors (**ii**) and (**v**) (green and yellow, respectively).

To explain the reason for preferential and higher selectivity of 2-amino-5-nitrothiazole derived semicarbazones towards MAO-B, the docked conformation of the representative lead compound of the series, compound **4**, was aligned with the docked conformations of our previously reported lead MAO-B inhibitors (**i**) and (**ii**). It was observed that the small hydrophobic ring of compound **4** (5-nitrothiazole moiety, site A) was more closer to FAD in comparison to the larger and bulkier hydrophobic rings of the inhibitors (**i**) and (**ii**) (6-nitrobenzothiazole and methylene(dioxy) moieties, respectively) when binding to the MAO-B active site (Figure 10(A)) which resulted in increased vander Waals interactions between compound **4** and FAD and hence attributed to the higher selectivity towards MAO-B.

Comparison of the experimental and virtual binding modes of the lead AChE inhibitor of the present series, compound **21** with our previously identified lead AChE inhibitors (**ii**)^50^ and (**v**) (unpublished) indicated that the presence of small 5-nitrothiazole moiety resulted in the decrease in the AChE inhibitory activity both experimentally and computationally when compared to the compounds possessing larger and bulky hydrophobic rings as in case of the inhibitors (**ii**) and (**v**) (methylene(dioxy) and 6-nitrobenzothiazole moieties, respectively). The higher potency of inhibitors (**ii**) and (**v**) may be because of the linear conformation adopted by them which allowed them to span across CAS and PAS cavities of AChE thereby contributing to their superior binding towards AChE. Whereas, inhibitor **21** adopted a non-linear conformation which eventually resulted in decreased potency and affinity (Figure 10(B)).

### *In vitro* antioxidant activity (DPPH radical scavenging assay)

The antioxidant activity data of the selected test compounds are listed in Table 5. The DPPH^•^ radical was scavenged by antioxidants *via* donation of hydrogen resulting in the formation of DPPH-H^•^. The colour of the DPPH changed from purple to yellow after reduction, which was quantified by the decline of absorbance at a wavelength of 517 nm. Among the tested compounds, **21** showed better antioxidant activity than ascorbic acid (Table 5). The results indicated that the presence of bromo group is critical in determination of scavenging activity.

**Table 5.** Antioxidant activity data of selected compounds.

Compound	% inhibition	Compound	% inhibition
**4**	58.68	**14**	55.29
**5**	51.83	**15**	10.58
**6**	30.43	**18**	10.77
**8**	11.60	**19**	24.03
**9**	35.89	**20**	47.16
**12**	42.56	**21**	65.65
**13**	39.39	**Ascorbic acid**	61.24

The statistical significance was calculated by one-way ANOVA followed by Dunnett’s test. *p <* .05 when compared with control.

### Neurotoxicity screening (rotarod test)

Selected compounds were screened for neurotoxicity by rotarod apparatus at a dose of 30 mg/kg at four time intervals viz. 0.5, 1, 2, and 4 h. The results are presented in Table 6. All the tested compounds were found to be non-neurotoxic except compounds **9** and **21**. Compound **9** was found to be moderately neurotoxic while compound **21** was mildly neurotoxic relative to reference drug phenytoin.

**Table 6.** Neurotoxicity screening results of selected compounds.

Compound	Neurotoxicity (*t* (h))^a^			
	0.5	1	2	4
**4**	0/4	0/4	0/4	0/4
**5**	0/4	0/4	0/4	0/4
**7**	0/4	0/4	0/4	0/4
**9**	2/4	1/4	0/4	0/4
**12**	0/4	0/4	0/4	0/4
**13**	0/4	0/4	0/4	0/4
**16**	0/4	0/4	0/4	0/4
**18**	0/4	0/4	0/4	0/4
**19**	0/4	0/4	0/4	0/4
**20**	0/4	0/4	0/4	0/4
**21**	1/4	0/4	0/4	0/4
**Phenytoin**	0/4	0/4	0/4	0/2

a Values are the number of animals that exhibit toxicity out of the total number of animals tested.

## Conclusions

Herein, we reported the design, synthesis, evaluation, and molecular docking of some 2-amino-5-nitrothiazole derived semicarbazones (**4**–**21**) as multi-targeted compounds with anti-ChE and MAO inhibition properties for the treatment of NDDs. Most of the compounds were found to exhibit inhibitory potencies in the micromolar to sub-micromolar range. The compounds were found to be selectively active against MAO-B isoform, thus indicating that thiazole moiety could be an interesting scaffold for the design of selective MAO-B inhibitors. Compound **4**, 1-(1-(4-bromophenyl)ethylidene)-4-(5-nitrothiazol-2-yl)semicarbazide, emerged as the lead MAO-B inhibitor, which ranked at the top in both experimental MAO inhibition assay (IC_50_ = 0.212 ± 0.004 µM, SI = 331.08) and computational molecular docking studies (*K*_i_ = 13.84 µM). Further kinetic studies indicated that compound **4** was competitive and reversible against MAO-B. To test the multifunctional behaviour of these compounds, they were also explored for AChE and BuChE inhibitory activity. In agreement with the experimental IC_50_ data, compound **21**, 1–(5-bromo-2-oxoindolin-3-ylidene)-4–(5-nitrothiazol-2-yl)semicarbazide (IC_50_ = 0.2645 ± 0.009 µM, mixed and reversible) showed the best binding energy value (*ΔG* = −6.71 kcal mol^−1^) for the rhAChE, and hence considered to be lead inhibitor for AChE, whereas compound **17** 1-((4-chlorophenyl)(phenyl)methylene)-4-(5-nitrothiazol-2-yl)semicarbazide (IC_50_ =  0.024 ± 0.002 µM, mixed and reversible) showing the best binding energy value (*ΔG* = −6.99 kcal mol^−1^) was found to be lead BuChE inhibitor. Examination of the various interactions observed during molecular docking studies pointed out the crucial residues accountable for appropriate binding of ligand to both the MAO isozymes and ChEs (AChE and BuChE). Computational docking studies revealed the importance of a flexible semicarbazino (–NH–CO–NH–N = C<) linker possessing hydrogen bonding regions in directing the optimal orientation of the hydrophobic heteroaryl residue at amino terminal (5-nitrothiazole moiety) and aryl/heteroaryl residue at the carbimino terminal into their respective binding pockets within the active site of MAO isozymes (MAO-A and MAO-B) and ChEs (AChE and BuChE). Additionally, the presence of bromo group on the hydrophobic aryl/heteroaryl ring at the carbimino terminal of semicarbazone template was essential for effective binding and stabilisation within the active site cavity of both the MAO isozymes and AChE. Moreover, compound **4** was found to be non-neurotoxic and presented good antioxidant activity although slightly less compound ascorbic acid. However, compound **21** showed slightly neurotoxic profile but was found to exhibit better antioxidant potential compared to ascorbic acid in DPPH radical scavenging assay.

Thus, replacement of 6-nitrobenzothiazole moiety with 5-nitrothiazole moiety at the amino terminal of SCZ template (site A) caused a substantial decrease in the activity toward MAO-A but either retained or slightly decreased potency and increased selectivity toward MAO-B.

This study revealed the requirement of a small heteroaryl ring at the amino terminal of semicarbazone template for the preferential inhibition and selectivity towards MAO-B. Our results suggest that 5-nitrothiazole derived semicarbazones could be further exploited for its potential multi-targeted role in the discovery of new drugs against NDDs.
